# Seroprevalence of Chronic Hepatitis B Virus Infection and Prior Immunity in Immigrants and Refugees: A Systematic Review and Meta-Analysis

**DOI:** 10.1371/journal.pone.0044611

**Published:** 2012-09-05

**Authors:** Carmine Rossi, Ian Shrier, Lee Marshall, Sonya Cnossen, Kevin Schwartzman, Marina B. Klein, Guido Schwarzer, Chris Greenaway

**Affiliations:** 1 Centre for Clinical Epidemiology and Community Studies of the Lady Davis Institute for Medical Research, Jewish General Hospital, Montreal, Canada; 2 Department of Epidemiology, Biostatistics & Occupational Health, McGill University, Montreal, Canada; 3 Respiratory Epidemiology and Clinical Research Unit, Montreal Chest Institute, McGill University, Montreal, Canada; 4 Division of Infectious Diseases, McGill University Health Centre, McGill University, Montreal, Canada; 5 Institute of Medical Biometry and Medical Informatics, University of Freiburg, Freiburg, Germany; 6 Division of Infectious Diseases, Jewish General Hospital, McGill University, Montreal, Canada; Centers for Disease Control and Prevention, United States of America

## Abstract

**Background:**

International migrants experience increased mortality from hepatocellular carcinoma compared to host populations, largely due to undetected chronic hepatitis B infection (HBV). We conducted a systematic review of the seroprevalence of chronic HBV and prior immunity in migrants arriving in low HBV prevalence countries to identify those at highest risk in order to guide disease prevention and control strategies.

**Methods and Findings:**

Medline, Medline In-Process, EMBASE and the Cochrane Database of Systematic Reviews were searched. Studies that reported HBV surface antigen or surface antibodies in migrants were included. The seroprevalence of chronic HBV and prior immunity were pooled by region of origin and immigrant class, using a random-effects model. A random-effects logistic regression was performed to explore heterogeneity. The number of chronically infected migrants in each immigrant-receiving country was estimated using the pooled HBV seroprevalences and country-specific census data. A total of 110 studies, representing 209,822 immigrants and refugees were included. The overall pooled seroprevalence of infection was 7.2% (95% CI: 6.3%–8.2%) and the seroprevalence of prior immunity was 39.7% (95% CI: 35.7%–43.9%). HBV seroprevalence differed significantly by region of origin. Migrants from East Asia and Sub-Saharan Africa were at highest risk and migrants from Eastern Europe were at an intermediate risk of infection. Region of origin, refugee status and decade of study were independently associated with infection in the adjusted random-effects logistic model. Almost 3.5 million migrants (95% CI: 2.8–4.5 million) are estimated to be chronically infected with HBV.

**Conclusions:**

The seroprevalence of chronic HBV infection is high in migrants from most world regions, particularly among those from East Asia, Sub-Saharan Africa and Eastern Europe, and more than 50% were found to be susceptible to HBV. Targeted screening and vaccination of international migrants can become an important component of HBV disease control efforts in immigrant-receiving countries.

## Introduction

Hepatitis B virus (HBV) infection is an important global health problem. Approximately 350 million people are chronically infected with the virus worldwide, 25% of whom will die from long term sequelae, such as cirrhosis, liver failure and hepatocellular carcinoma (HCC), resulting in 600,000 to one million deaths annually [1], [2]. Morbidity and mortality from hepatitis B can be reduced through screening individuals at risk for chronic HBV infection, and offering appropriately timed antiviral therapy to those found to be positive [3]. Furthermore, an effective HBV vaccine to protect those who are susceptible to HBV has been available since the 1980s in most high income countries [4], [5].

Over the past four decades, international migration has increased at an unprecedented rate and the majority of new migrants arriving in low hepatitis B prevalence countries [hepatitis B surface antigen (HBsAg) seroprevalence <2%] have originated from intermediate (HBsAg seroprevalence between 2%–7%) or high hepatitis B prevalence countries (HBsAg seroprevalence ≥8%) [6]. During this time period the incidence of chronic HBV infection and incidence and mortality rates of HCC in North America and Western Europe have increased, likely due in part to undetected chronic HBV infection in the migrant population [7], [8]. This is supported by the fact that migrants have both higher incidence of chronic HBV infection and HCC and increased mortality from cirrhosis and HCC, compared to host populations [9]–[13].

Despite these disparities, immigrants and refugees from intermediate and high HBV prevalence countries are not routinely screened for HBV infection, nor is hepatitis B vaccination routinely given after arrival in most immigrant-receiving countries. We conducted a systematic review and meta-analysis of chronic HBV infection and prior immunity in the migrant population to identify groups at highest risk in order to guide disease prevention and control strategies in immigrant-receiving countries.

## Methods

### Search Strategy and Study Selection

This review was prepared in accordance with PRISMA guidelines [14]. The systematic review research protocol is available online with the supporting information (See Text S1). Medline, Medline In-Process, EMBASE, and the Cochrane Database of Systematic Reviews were searched for studies reporting the seroprevalence of chronic HBV infection and immunity in immigrants and refugees, using no initial language restrictions, from the earliest date until November 1^st^, 2011. The search strategy that was used for all databases is shown in Table 1. Additional studies were identified by examining the reference lists of eligible studies and the bibliography of review articles. The titles and abstracts of all identified articles were reviewed by two authors. For those studies that met the pre-defined eligibility criteria, full-text articles were obtained and reviewed by two authors. Reasons for exclusion were recorded for all full-text articles. Original studies published in English, French or Italian that reported data on the seroprevalence of chronic HBV infection and/or HBV immunity in immigrants or refugees originating from intermediate or high HBV prevalence countries and arriving in low HBV prevalence countries were included. Only studies that recruited migrants who were representative of the general migrant population were included. Studies that focused on migrant groups at increased risk for hepatitis B due to certain behaviours were excluded (i.e. sex workers, injection drug users, etc…) [15], [16].

**Table 1:** Systematic review search strategy.

1	exp Hepatitis B/
2	(hepatitis b or hepatitis b virus or chronic hepatitis b or hbv or chb).tw.
3	1 or 2
4	exp “Emigration and Immigration”/
5	(resettlement or re-settlement or border crossing or newcomer or naturalized citizen or nonnative or settler or new arrival or displacedperson or in-migration or migration or migrant or immigrant or immigration or emigrant or emigration).tw.
6	4 or 5
7	exp Refugees/
8	(asylum seeker or refugee or displaced person or alien).tw.
9	7 or 8
10	3 and (6 or 9)

### Data Extraction

Data were extracted independently and in duplicate, using a piloted and standardized data extraction form, by two authors for all articles in English or French. For the one study reviewed that was published in Italian, data was extracted by one author. Data were then entered independently and in duplicate into a Microsoft Access Database and imported into SAS (version 9.2, Cary, North Carolina) for comparing, cleaning and analysis. Discrepancies were corrected by consensus.

### Study Outcomes and Variables

The primary study outcomes were 1) the proportion of subjects with chronic HBV infection among those screened and 2) the proportion of subjects with HBV immunity among those screened. Chronic HBV infection was defined as the presence of HBsAg, which is a serologic marker of either acute or chronic HBV infection. We assumed the presence of HBsAg represented imported chronic HBV infections rather than a new acute infection, given the fact that most immigrants and refugees from intermediate and high HBV prevalence countries acquire HBV infection during the perinatal period or in early childhood [5]. HBV immunity was defined as the presence of hepatitis B surface antibody (anti-HBs), with or without hepatitis B core antibody (anti-HBc). Anti-HBs are serologic markers that appear in individuals who have resolved an acute HBV infection and may also be found in individuals who have been immunized.

Data on study design, decade of study, country of landing, immigrant status, migrants’ region of origin, mean or median age, gender distribution, co-morbidities, method of participant identification for study, and serologic testing method used were also extracted. The method of participant selection was categorized as occurring in reception centers at the time of arrival, in the context of a clinic or hospital visit, screening of pregnant women or other situations [i.e. screening studies in general host populations that included a subset of migrants (i.e. the army, schools or community surveys) or studies which invited certain immigrant groups to be screened]. Data on immigrant status was classified into the following categories: immigrants, refugees, asylum seekers, and adopted children. A dichotomous variable of immigrant status combined immigrants and adopted children into ‘immigrants’ and refugees and asylum seekers into ‘refugees’, because of the very small number of studies with adopted children or asylum seekers. Region of origin was classified according to the World Bank country classification and included the following regions: Latin America and the Caribbean, Eastern Europe and Central Asia, Middle East and North Africa, Sub-Saharan Africa, South Asia, and East Asia and the Pacific [17]. If a study contained migrants from more than one region, then seroprevalences for infection and immunity were obtained separately for each region of origin, if possible.

### Statistical Analysis

For each study, the seroprevalence of chronic HBV infection and HBV immunity was calculated by using the reported numbers of subjects positive for HBsAg and anti-HBs serologic markers, respectively, divided by the total number of people screened for each of these markers. Proportions were transformed with the logit transformation and pooled using a random-effects model to account for the expected high heterogeneity between studies [18]. The logit-transformed proportions were back-transformed and results were presented as percentages. The logit transformation was used to avoid studies with few events from being weighted too heavily in the random-effects model [19] and because the multivariate analysis, which used a random-effects logistic regression model, is also based on the logit transformation (see below). Overall heterogeneity among studies was assessed using the *I* ^2^ statistic and estimates were stratified by region of origin and immigration status, as these variables were believed *a priori* to be important predictors of HBV infection [20]. The data were not stratified by age or gender as few studies, 40% and 26% respectively, included any form of information on these variables. The meta-analysis was performed using the *metaprop* command of the meta package in R (version 2.13.1) [21].

A random-effects logistic regression, using the *glmer* command of the lme4 package to fit generalized linear mixed-models in R, was used to further explore heterogeneity and to compare the odds of being infected among migrants from the different regions of origin after adjusting for potential confounding by immigrant status and decade of study [22]. Three studies that did not report estimates separately for immigrants and refugees and 18 studies that did not report estimates separately by region of origin were excluded from the random-effects logistic regression model.

To estimate the number of migrants who are chronically infected with hepatitis B in immigrant-receiving countries, data on the number of foreign-born residents from different regions of origin living in host countries was obtained from the national statistical agencies for each of these countries (see Appendix Table 1 in Text S2). This data and the pooled region-specific seroprevalence of chronic HBV infection in immigrants and refugees estimated in this review were multiplied to estimate the burden of chronic HBV infection in migrants living in traditional immigrant-receiving countries.

## Results

### Search Results

**Figure 1:**
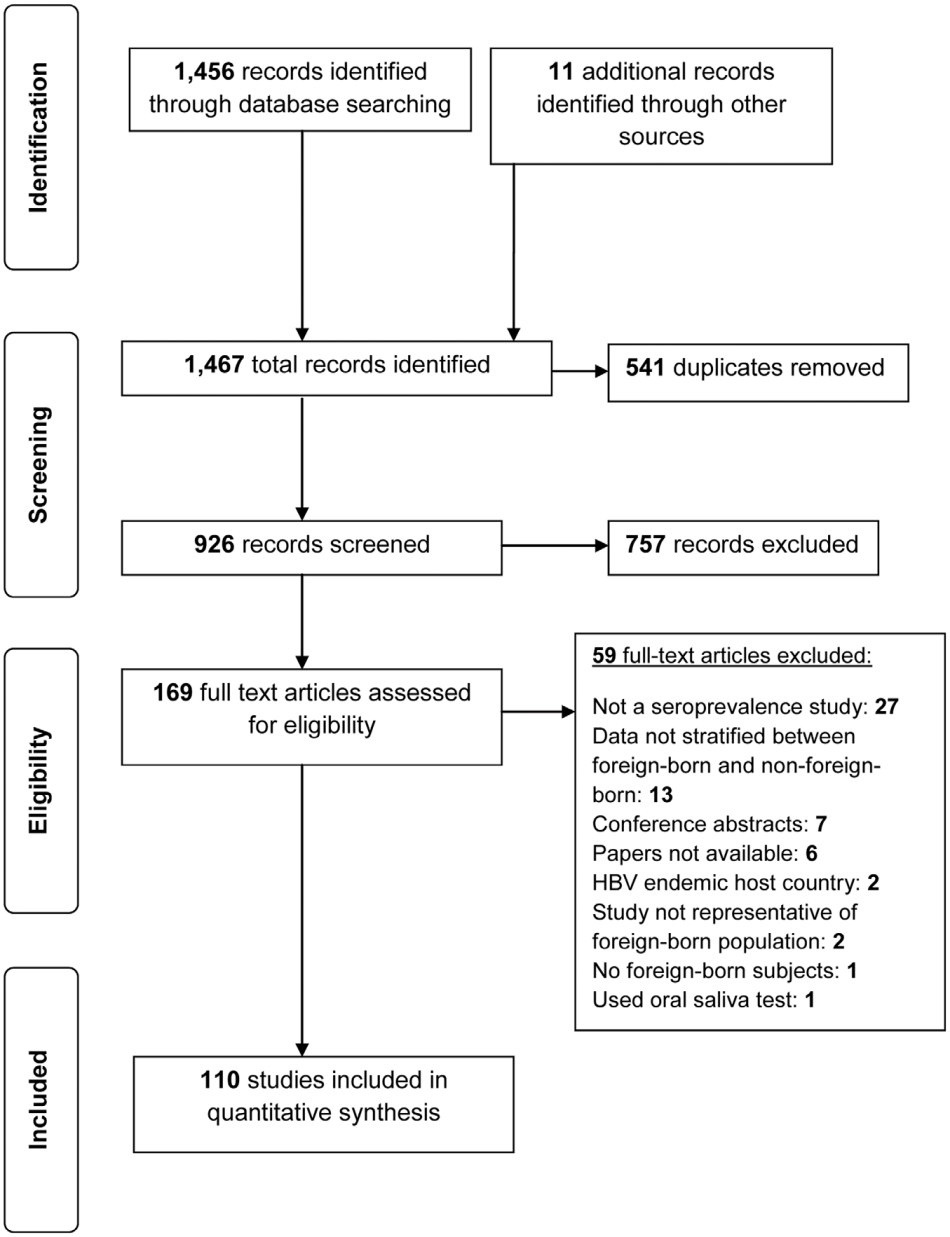
Summary of literature search and study selection.

A total of 1,456 citations were identified in the electronic search and an additional 11 citations were identified through hand searching (Figure 1). After duplicates were removed, 926 citations were screened with the title and abstract and 757 were excluded. A total of 169 articles were assessed with predefined eligibility criteria in the full-text review and 53 were excluded. An additional six articles were excluded because the full-text could not be retrieved despite assistance from a McGill University librarian. A total of 110 studies were included in the systematic review and meta-analysis (Appendix Table 2 in Text S2).

**Table 2:** Pooled seroprevalence of chronic hepatitis B infection by immigrant class and region of origin.

		Number of Studies	Number of Subjects	Pooled HBsAg Seroprevalence Percent (95% CI)	*I* ^2^ (%)
Overall Seroprevalence	Overall	110	209,822	7.2 (6.3 to 8.2)	98.1
	Immigrants	50	72,510	5.1 (4.0 to 6.4)	97.0
	Refugees	57	134,418	9.6 (8.2 to 11.1)	98.1
	Mixed	3	2,894	7.7 (6.7 to 8.7)	0
East Asia & The Pacific	Overall	40	62,258	11.3 (10.3 to 12.4)	89.1
	Immigrants	16	8,550	8.6 (6.7 to 10.9)	89.2
	Refugees	23	53,381	13.2 (12.0 to 14.4)	85.8
	Mixed	1	327	8.6 (6.0 to 12.1)	0
Sub-Saharan Africa	Overall	33	22,219	10.3 (9.1 to 11.8)	86.2
	Immigrants	12	4,581	9.9 (7.1 to 13.7)	90.3
	Refugees	19	16,022	10.5 (9.0 to 12.2)	84.3
	Mixed	2	1,616	10.7 (8.1 to 13.9)	65.5
Eastern Europe & Central Asia	Overall	37	31,549	5.8 (4.3 to 7.9)	96.8
	Immigrants	13	14,301	5.9 (4.1 to 8.5)	93.4
	Refugees	22	17,169	5.9 (3.7 to 9.1)	97.5
	Mixed	2	79	6.6 (0.8 to 37.2)	63.5
South Asia	Overall	11	1,567	4.6 (2.6 to 7.8)	71.5
	Immigrants	4	441	2.4 (0.3 to 15.4)	73.3
	Refugees	5	845	6.5 (3.8 to 11.1)	71.9
	Mixed	2	281	2.0 (0.9 to 4.5)	0
Middle East & North Africa	Overall	17	19,127	2.0 (1.6 to 2.9)	79.0
	Immigrants	8	12,541	1.8 (1.3 to 2.7)	79.8
	Refugees	7	6,470	2.6 (1.3 to 4.9)	84.7
	Mixed	2	116	3.6 (1.4 to 9.2)	0
Latin America & Caribbean	Overall	18	29,554	1.7 (1.1 to 2.7)	86.1
	Immigrants	9	9,539	1.4 (0.8 to 2.7)	77.2
	Refugees	7	19,580	3.1 (0.8 to 11.6)	92.3
	Mixed	2	435	0.6 (0.2 to 2.0)	0

The total number of subjects for each specific region exceeds those reported in Appendix Table 3 because they include available data from studies that included mixed populations in terms of region of origin. Proportions were logit transformed prior to pooling with a random-effects model. HBsAg  =  hepatitis B surface antigen. CI  =  confidence interval.

### Seroprevalence of Chronic Hepatitis B Infection in Migrants

All 110 studies reported the seroprevalence of HBV infection in immigrants or refugees, representing a total of 209,822 migrants from all world regions. Just over half of the seroprevalence studies of chronic HBV infection included refugees and asylum seekers and nearly a quarter studied migrants exclusively from East Asia and the Pacific, although almost half of all the studies screened migrants from more than one region (Appendix Table 3 in Text S2). All studies were conducted in the context of asymptomatic screening and almost 90% of study participants were recruited in settings with low risk for selection bias (i.e. mass screening in reception centers or among pregnant women). The number of studies published on the seroprevalence of HBV infection increased every decade after the initial studies first reported on the seroprevalence of infection in refugees from Southeast Asia.

**Table 3:** Unadjusted and adjusted random-effects logistic regression of chronic hepatitis B infection.

Variable		Number of Studies	Unadjusted OR (95% CI)	*P* Value	Adjusted OR (95% CI)	*P* Value
Immigrant Status	Immigrant	36	Reference		Reference	
	Refugee	53	1.71 (1.18 to 2.49)	0.005	1.42 (1.01 to 1.99)	0.042
Region of Origin^a^	Latin America	16	Reference		Reference	
	Eastern Europe	35	2.32 (1.99 to 2.69)	<0.001	2.29 (1.97 to 2.67)	<0.001
	Middle East	15	1.34 (1.14 to 1.58)	<0.001	1.34 (1.14 to 1.58)	<0.001
	Sub-Saharan Africa	31	6.71 (5.84 to 7.71)	<0.001	6.68 (5.81 to 7.68)	<0.001
	South Asia	9	3.72 (2.72 to 5.10)	<0.001	3.76 (2.75 to 5.15)	<0.001
	East Asia	39	10.8 (9.45 to 12.3)	<0.001	10.8 (9.44 to 12.3)	<0.001
Decade of Study	1980s	29	Reference		Reference	
	1990s	24	0.81 (0.50 to 1.32)	0.40	1.58 (1.03 to 2.43)	0.035
	2000s	36	0.59 (0.38 to 0.92)	0.02	1.17 (0.80 to 1.74)	0.41

Three studies of the 110 total studies were dropped because they did not report separate estimates for refugees and immigrants, and a further 18 studies were dropped because they did not report separate estimates for the different region of origins within that study. A total of 89 studies were included in the random-effects logistic regression. CI  =  Confidence Interval. OR  =  Odds Ratio.

a The sum of the total number of studies for each origin is greater than 89 because several studies reported more than one origin.

The overall pooled seroprevalence of infection in all international migrants, among the 110 studies, was 7.2% (95% CI: 6.3%–8.2%). Higher seroprevalences were found among refugees and asylum seekers, compared immigrants (Table 2). Pooled HBsAg seroprevalence estimates by region of origin differed from one another and were similar to global estimates from the Centers for Disease Control and Prevention (CDC), the World Health Organization (WHO) and a recent systematic review of global seroprevalence of HBV infection [23]–[25]. These organizations divide the world into high (HBsAg seroprevalence ≥8%), intermediate (HBsAg seroprevalence between 2%–7%) and low (<2%) chronic hepatitis B endemic regions. Our region-specific pooled proportions for migrants fell within the same ranges for the HBV seroprevalence categories defined by the WHO and CDC (see Figure S1). Chronic HBV seroprevalence was high for migrants from East Asia and the Pacific and from Sub-Saharan Africa [11.3% (95% CI: 10.3%–12.4%) and 10.3% (95% CI: 9.1%–11.8%), respectively], intermediate for migrants from Eastern Europe and Central Asia and from South Asia [5.8% (95% CI: 4.3%–7.9%) and 4.6% (95% CI: 2.6%–7.8%), respectively], and low for migrants from Latin America and the Caribbean and the Middle East and North Africa [1.7% (95% CI 1.1%–2.7%) and 2.0% (95% CI: 1.6%–2.9%), respectively].

Results from the random-effects logistic regression analysis showing both unadjusted and adjusted odds ratios of chronic HBV infection are reported in Table 3. Different models, with and without interaction terms, using traditional model selection criteria were explored using region of origin, immigrant status and decade of study as variables. The best model without interaction terms, as measured with the lowest *Akaike information criterion* (AIC) value, included only region of origin, however models including immigrant status and decade of study were very similar (odds ratio differences by ≤0.06). Models that included interactions between variables were also explored. Although there were some statistically significant interactions in some models, there was no consistent significant trend of interactions across models. The model with all three of these variables without interactions was chosen as we felt that this was the most relevant for clinicians working with migrant populations. In the final model, migrants from all other world regions had significantly greater odds of being chronically infected with HBV as compared to migrants from Latin America and the Caribbean, the region with the lowest seroprevalence and the one most closely approximating the estimated prevalence in immigrant-receiving countries, after adjusting for immigrant status and decade of study. Refugees were found to have 1.42 (95% CI: 1.01–1.99) greater odds of being chronically infected compared to immigrants, after adjustment for region of origin and decade of study. Migrants in studies from the 1990s were found to have 1.58 (95% CI: 1.03–2.43) greater odds of being chronically infected, compared to migrants from studies reported in the 1980s, after adjusting for the other study-level covariates.

### Seroprevalence of Hepatitis B Immunity in Migrants

Of the 110 studies included in this systematic review, 39 reported data on the seroprevalence of HBV immunity in immigrants or refugees and included data on 40,330 migrants from all world regions. As with studies reporting the seroprevalence of chronic HBV infection, just over half of the studies examining HBV immunity screened refugees (Appendix Table 4 in Text S2). The overall pooled seroprevalence of immunity to HBV was 39.7% (95% CI: 35.7%–43.9%) and was highest in those regions that had the highest seroprevalence of chronic HBV infection (Table 4). Half of the migrants from East Asia & the Pacific [50.2% (95% CI: 45.8%–54.6%)] and almost half of Sub-Saharan African migrants [41.7% (95% CI: 37.6%–45.9%)] were immune to HBV. The seroprevalence of immunity was lower in regions where the seroprevalence of chronic HBV infection was lowest. However, the number of subjects screened for anti-HBs from the Middle East and North Africa (n = 131), South Asia (n = 364) and Latin America and the Caribbean (n = 29) were very small and results from these regions were imprecise. There were too few studies to examine differences in immunity between immigrants and refugees from each specific region of origin.

**Table 4:** Pooled seroprevalence of hepatitis B immunity by region of origin and immigrant class.

		Number ofStudies	Number ofSubjects	Pooled anti-HBs SeroprevalencePercent (95% CI)	*I* ^2^ (%)
Overall Seroprevalence	Overall	39	40,330	39.7 (35.7 to 43.9)	98.1
	Immigrants	16	7,207	33.7 (25.1 to 43.5)	98.3
	Refugees	22	32,796	41.5 (37.1 to 46.0)	97.9
	Mixed	1	327	76.5 (71.5 to 81.0)	0
East Asia & The Pacific		14	19,204	50.2 (45.8 to 54.6)	92.3
Sub-Saharan Africa		7	1,120	41.7 (37.6 to 45.9)	45.3
Eastern Europe & Central Asia		12	8,207	30.1 (21.8 to 39.9)	98.6
South Asia		2	364	10.6 (5.9 to 18.3)	71.7
Middle East & North Africa		2	131	24.3 (10.7 to 46.2)	31.7
Latin America & Caribbean		2	29	33.0 (5.8 to 79.8)	55.4

The total number of subjects for each specific region exceeds those reported in Appendix Table 4 because they include available data from studies that included mixed populations in terms of region of origin. Proportions were logit transformed prior to pooling with a random-effects model. Anti-HBs  =  hepatitis B surface antibody. CI  =  confidence interval.

### Burden of Chronic HBV Infection in Migrants

Almost 3.5 million immigrants and refugees (95% CI: 2.8–4.5) living in immigrant-receiving countries were estimated to be chronically infected with HBV (Table 5 and Figures S2 and S3). The proportion of all migrants chronically infected ranged from 3.7% to 9.7% in the different host countries with the largest estimated number residing in the United States (1.6 million), Canada (285,000), Germany (284,000), Italy (201,000), the United Kingdom (193,000), and Australia (176,000).

**Table 5:** Country-specific estimates of the burden of chronic hepatitis B infection in migrants in traditional immigrant-receiving countries.

		Number of Immigrants	Estimated Number ofInfected Immigrants	Percent of Immigrantswith Chronic HBV
**North America**	Canada	4,271,500	285,000	6.7
	United States	35,500,500	1,607,000	4.5
**Europe**	Austria	993,000	58,000	5.8
	Belgium	411,000	22,500	5.5
	Czech Republic	390,000	26,500	6.8
	Denmark	286,000	16,500	5.8
	Finland	181,000	11,500	6.4
	France	2,348,000	113,500	4.8
	Germany	4,784,000	284,000	5.9
	Greece	684,000	39,000	5.7
	Israel	1,148,000	54,000	4.7
	Italy	3,684,500	201,500	5.5
	Netherlands	1,395,000	73,500	5.3
	Norway	395,000	25,500	6.5
	Portugal	385,500	22,500	5.8
	Republic of Ireland	247,000	17,000	6.9
	Spain	3,487,000	128,500	3.7
	Sweden	965,500	52,500	5.4
	Switzerland	691,000	41,000	5.9
	United Kingdom	3,002,000	193,500	6.4
**Oceania**	Australia	2,141,000	176,000	8.2
	New Zealand	491,500	47,500	9.7

The number of immigrants was obtained from recent census data from all immigrant-receiving countries and then rounded (See Appendix Table 1 in Text S2). HBV  =  hepatitis B virus.

## Discussion

Results from this systematic review and meta-analysis highlight the fact that migrants originating from intermediate or high hepatitis B prevalence countries who live in low HBV prevalence immigrant-receiving countries are an important risk group for chronic HBV infection. The pooled seroprevalence estimates of chronic hepatitis B in migrants from different world regions mirrored the prevalence of chronic HBV in their regions of origin. We found that migrants from East Asia and the Pacific and Sub-Saharan Africa had the highest seroprevalence of chronic HBV infection (≥10% HBsAg positive), migrants from Eastern Europe and Central Asia and South Asia had intermediate seroprevalence (4%–6%) and those from the Middle East and North Africa and Latin America and the Caribbean regions had low seroprevalence (≤2%). Our estimates of the proportion of immigrants with chronic HBV infection living in different immigrant-receiving countries in our study ranged from 3.7% to 9.7%. This data suggests that a large proportion of immigrants would benefit from screening for chronic HBV infection given that recent studies have shown that it is cost-effective to screen for chronic HBV infection at a seroprevalence as low as 1% [26]–[29]. Furthermore, over half of all migrants were found to be susceptible to HBV and could benefit from HBV immunization programs.

Another important finding of our review was that pooled estimates of chronic HBV seroprevalence were higher in refugees compared to immigrants. This trend was seen in all world regions except for migrants from Eastern Europe and Central Asia. In addition, refugees were found to have 42% greater odds of being chronically infected with HBV as compared to immigrants after adjusting for region of origin and decade of study in the random-effects logistic model. This is plausible given the fact that refugees may have experienced violent acts that put them at increased risk of exposure to infected blood through horizontal or sexual transmission [30], [31]. Although the effect of refugee status on HBV infection was significant after adjusting for region of origin and study decade, these results need to be interpreted with caution as age and gender were infrequently reported in the source studies and may have confounded the association between refugee status and infection.

We estimate that nearly three and half million migrants living in low HBV prevalence countries are chronically infected with HBV, with the largest burden occurring in the United States (1.6 million), Canada (285,000) and Germany (284,000). The burden of HBV infection in immigrants and refugees is significantly higher than in other high-risk groups, such as injection drug users, in whom a recent review reported that just over one million were chronically infected HBV worldwide [32]. In addition immigrants have a higher incidence of HCC and associated mortality as compared to host populations in large part likely due to undetected HBV infection [9], [33]. Despite this health disparity immigrants have not traditionally been among the identified groups at high risk for HBV and consequently are not routinely screened prior to or after arrival in low HBV prevalence immigrant-receiving countries [34]. Recent guidelines from the United States, Canada, the United Kingdom and Australia however serve to raise awareness that immigrants are an important group at risk for HBV who should be screened for HBV [3], [35]–[37]. The strongest recommendations are from the CDC in the United States and the Canadian Collaboration for Immigrant and Refugee Health who both recommend that all immigrants originating from countries with a HBV seroprevalence greater than 2% should be screened for chronic HBV infection and prior immunity to HBV and vaccinated if found to be susceptible [3], [35].

The proportion of immigrants actual being screened however, remains low despite these recent recommendations, due to the fact that screening occurs most frequently on an ad hoc basis in the primary care setting [38], [39]. A recent report by the Institute of Medicine identified several gaps in opportunities to prevent and control of HBV. These were due to a lack of knowledge and awareness about chronic viral hepatitis on the part of health care and social service providers, at-risk populations, and members of the public and policymakers. As a consequence there is insufficient understanding about the extent and seriousness of this public health problem resulting in inadequate public resources being allocated to prevention, control and surveillance programs [40]. The degree of under detection of chronic HBV in the United States was shown in a recent study that found that although there was an estimated 1.4 to 2 million cases of chronic HBV, fewer than 50,000 people received prescriptions for HBV antiviral medication per year [41].

One of the major strengths of this systematic review was that it included a very large number of migrants arriving from all world regions over a 40-year period to several different immigrant-receiving countries. This study captured seroprevalence estimates from several different cohorts of migrants, including movements of refugees from Southeast Asia in the 1980s, immigrants and refugees from the Balkans and Eastern Europe who arrived in the first decade of the 21^st^ century and more recent movements of individuals from different countries in Sub-Saharan Africa. We believe this review encapsulated a representative cross-section of international migrants and provides valid estimates for the seroprevalence of HBV infection and immunity in the general immigrant and refugee populations.

The major limitation of this study was the lack of data on the age and gender of included subjects. Less than half the studies reported mean or median age, and only 26% reported gender. Consequently, we were not able to stratify the data by these variables nor were we able to adjust for them in the random-effects logistic model. This is important because children may have a lower seroprevalence of chronic HBV, compared to adults, due to less years of cumulative exposure. Without adjusting for age, we may have underestimated the seroprevalence of infection in adult migrants. In addition, males have higher rates of chronic HBV and HCC as compared to females, for reasons that are not entirely well understood [8]. Without information on the gender composition of the studied population, we are unsure if our pooled seroprevalence estimates may have over or underestimated the proportion of migrants who are truly infected. Furthermore, if a significant proportion of refugees were males or older adults, then these variables may be confounding the relationship between refugee status and infection we identified in our logistic model. In addition, some cases of acute HBV may have been misclassified as chronic HBV in our analysis, as HBsAg is also detected in acutely infected individuals. However, this is unlikely to have had a large impact on our findings, because as discussed, most HBV infections in foreign-born populations originate early in life and progress to a chronic infection.

In low HBV prevalence immigrant-receiving countries, international migrants from intermediate and high HBV prevalence countries are a large unrecognized risk group for chronic hepatitis B. On July 28^th^, 2011, the world celebrated the first WHO sponsored World Hepatitis Day. The objective of creating this day was to focus attention on the global health threat of hepatitis and to promote actions to confront it [42]. This represented a global call to action to all countries to identify all persons with undetected chronic HBV living within their borders. The results of our study serve to raise awareness and educate health care workers and policy makers that immigrants are an important group at risk for chronic HBV infection. Targeted HBV screening and vaccination programs for all groups at risk for HBV would result in early detection of HBV infection, decreased transmission to susceptible household members and decrease individual HBV-associated morbidity and mortality.

## Supporting Information

Figure S1 **Seroprevalence of chronic hepatitis B by region: Global classification and our estimates.**
(TIF)

Figure S2 **Estimated number of migrants with chronic hepatitis B infection living in North America and Oceania.**
(TIF)

Figure S3 **Estimated number of migrants with chronic hepatitis B infection living in Western Europe.**
(TIF)

Text S1 **Study protocol.**
(PDF)

Text S2 **Supplementary tables.**
(DOCX)
