# Supplementary figures and images for: Seroprevalence of Chronic Hepatitis B Virus Infection and Prior Immunity in Immigrants and Refugees: A Systematic Review and Meta-Analysis
Source: PLoS One. 2012 Sep 5;7(9):e44611. doi: 10.1371/journal.pone.0044611 (PMC3434171; doi:10.1371/journal.pone.0044611)

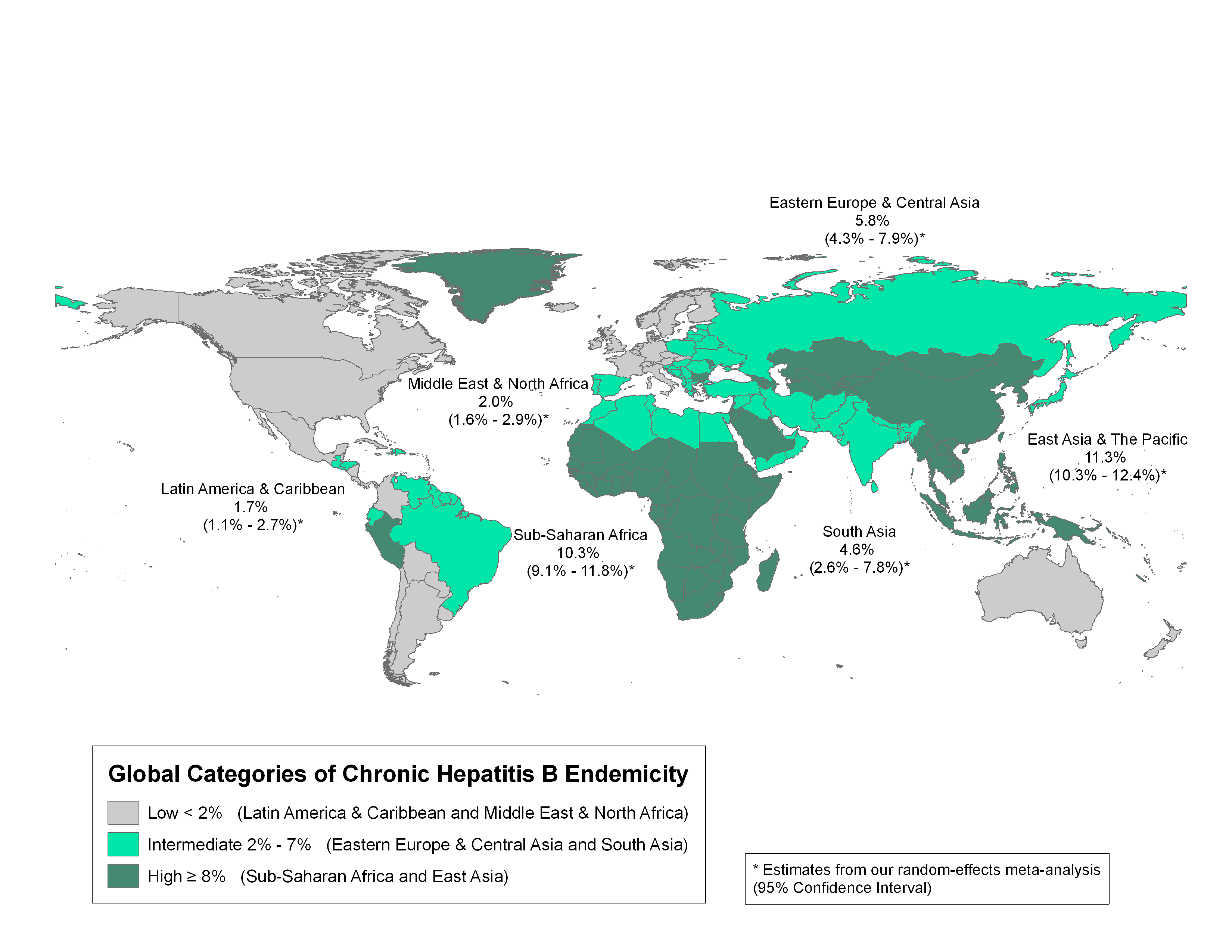

Supplement: Figure S1 — Seroprevalence of chronic hepatitis B by region: Global classification and our estimates. (TIF) [file pone.0044611.s001.tif]

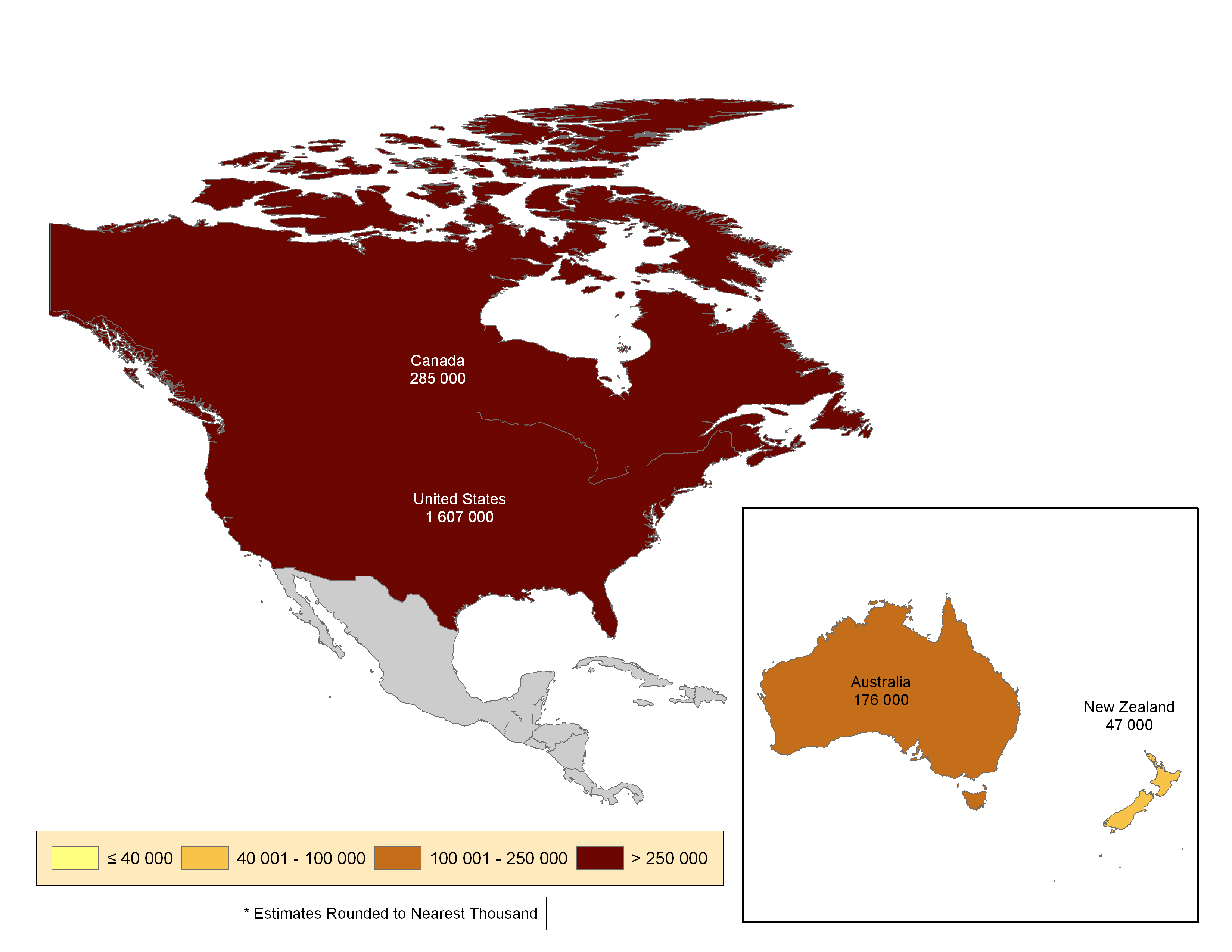

Supplement: Figure S2 — Estimated number of migrants with chronic hepatitis B infection living in North America and Oceania. (TIF) [file pone.0044611.s002.tif]

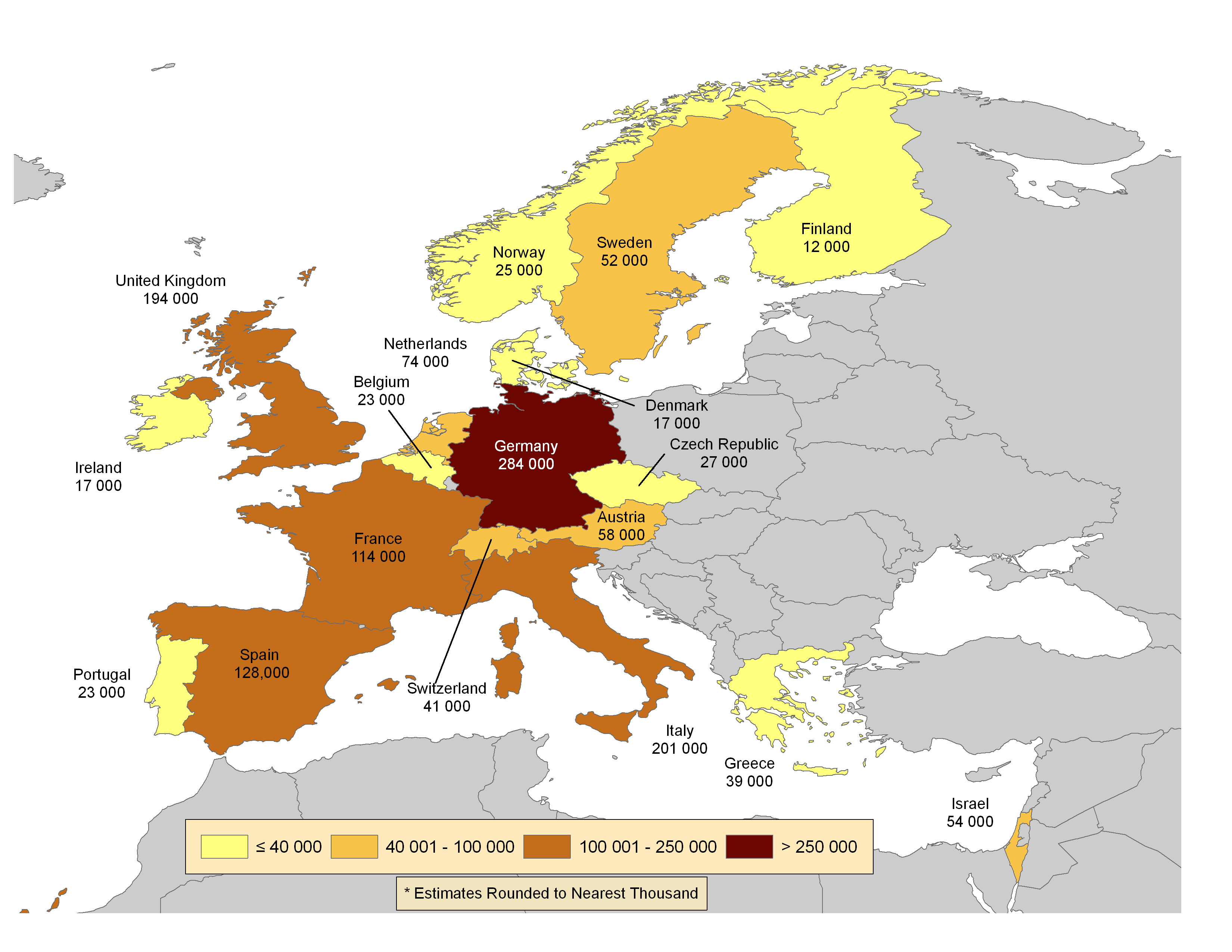

Supplement: Figure S3 — Estimated number of migrants with chronic hepatitis B infection living in Western Europe. (TIF) [file pone.0044611.s003.tif]
