# Supplementary material for: Seroprevalence of Chronic Hepatitis B Virus Infection and Prior Immunity in Immigrants and Refugees: A Systematic Review and Meta-Analysis
Source: PLoS One. 2012 Sep 5;7(9):e44611. doi: 10.1371/journal.pone.0044611 (PMC3434171; doi:10.1371/journal.pone.0044611)
Supplement: Text S1 — Study protocol. (PDF) [file pone.0044611.s004.pdf]

Seroprevalence of Hepatitis B Surface Antigen (HBsAg) and Hepatitis B immunity in the Immigrant and Refugee Population: A Systematic Review and Meta-Analysis

Rossi C, Marshall L, Cnossen S, Shrier I, Schwarzer G, Klein M, Schwartzman K, Greenaway C

1.0. BACKGROUND

Infection with the Hepatitis B Virus (HBV) is an important global health problem. It is estimated that 350 million people are currently infected with HBV, and nearly 1 million preventable deaths occur annually from HBV-related cirrhosis and hepatocellular carcinoma.[1] People chronically infected with hepatitis B have a 15%–25% lifetime risk of dying from cirrhosis and hepatocellular carcinoma.[1,2] They are typically asymptomatic until they present with end-stage liver disease or hepatocellular carcinoma several decades after infection. Canada is a country with low rates of hepatitis B and an overall seroprevalence of chronic hepatitis B infection of < 0.5%. Over the past 40 years however, most immigrants (> 70% of 250 000/yr) who arrived in Canada have originated from countries with intermediate (2-8% HepBsAg positive) or high (≥8% HepBsAg positive) rates of endemic hepatitis B. It is estimated that immigrants have an overall seroprevalence of chronic infection with hepatitis B of about 3% (0.5-20%) similar to rates in their countries of origin but this has not been systematically reviewed.[3,4]

Immigrant populations have higher mortality from chronic viral hepatitis and from hepatocellular carcinoma than the Canadian-born population. The majority of this burden is likely attributable to undetected chronic infection with hepatitis B. Treatment of chronic infection with hepatitis B decreases morbidity from chronic liver disease. Childhood hepatitis B vaccination programs decreases mortality from hepatocellular carcinoma and hepatitis B vaccination of adults reduces development of acute hepatitis B infection. Despite these interventions, there are no organized screening and treatment programs for chronic infection with hepatitis B for the immigrant population and they are not routinely offered hepatitis B vaccination outside of the universal childhood vaccination program. We propose to carry out a systematic review and meta-analysis to describe the prevalence of chronic hepatitis B infection and the prevalence of prior immunity to HBV among the immigrant populations in order to better understand groups at highest risk who would benefit from screening and treatment for chronic hepatitis B and/or hepatitis B vaccination. Information from this study will be used as input for a cost-effectiveness analysis on screening and vaccination for Hepatitis B in immigrants in Canada.

2.0. OBJECTIVES

2.1. Aim

a) To determine the prevalence of chronic hepatitis B infection (HBsAg positive) and prior immunity to Hepatitis B in the migrant population.

b) Stratify the above prevalence figures if possible by important predictors of chronic hepatitis B infection in the immigrant population such as immigration class and region of origin.

3.0. DEFINITIONS

HEPATITIS B

3.1. Hepatitis B Virus

Hepatitis B Virus (HBV) is a viral infection (double-stranded DNA virus) that causes acute and chronic infection of the liver. It is present in the blood and body fluids (semen, vaginal fluid, saliva) of an infected person. It is transmitted perinatally (infected mother to infant at the time of delivery), percutaneously (contaminated needles or equipment, unscreened blood products), sexually and within households (sharing personal care items contaminated with blood such as toothbrushes, razors, etc…). HBV is a vaccine-preventable disease (efficacy >85%) and it is important to identify those at risk who would benefit from vaccination in order to decrease HBV transmission. Chronic hepatitis B can be detected with widely available serologic tests and treatment can decrease the risk of developing the complication from chronic hepatitis B (cirrhosis, hepatocellular carcinoma). Chronic carriers serve as an important source of new infections; most have no signs or symptoms and an estimated two-thirds are unaware of their status.

3.2. Acute HBV Infection

An acute HBV infection may be asymptomatic, have non-specific symptoms or have frank symptomatic hepatitis, but resolves within six months of initial infection. After developing an acute infection, the likelihood of developing chronic HBV infection is inversely related to the age of acquisition of the infection. In infants infected at birth, 80-90% of them will develop a chronic HBV infection. In children infected between 1-4 years of age, 30-60% will develop a chronic infection. In immune-competent adults, < 10% will develop a long-standing infection.[5] Resolving the acute infection confers lifelong immunity on the host.

3.3. Chronic HBV Infection

Individuals who fail to clear the acute infection become chronic HBV carriers. Individuals chronically infected with HBV have a 15-25% lifetime risk of dying from cirrhosis and hepatocellular carcinoma (HCC).[1,2] HCC is one of the most fatal cancers, with a five-year relative survival rates less than 11% even in developed countries.[6] Chronic HBV infection is diagnosed by two positive HBsAg tests, six months apart (see serological markers below).

3.4 Immunity to HBV Infection

The presence of Hepatitis B surface antibody (Anti-HBs), alone, signifies immunity to the virus obtained from vaccination.

Presence of Hepatitis B Core Antigen (anti-HBc) combined with Hepatitis B surface antibody (Anti-HBs) also signifies immunity to the virus, obtained from resolving an acute infection.

SEROLOGIC MARKERS

HBsAg (surface antigen) indicates active infection. Persistence for 6 months indicates chronic infection, while clearance of this marker indicates recovery. Uncommonly it may be present at undetectable levels in chronic infection.

Anti -HBc IgM is a marker of early acute HBV infection, but may also reappear in chronic infection during flares of activity. Clinical/epidemiological correlation is required.

Anti-HBc (antibody to the core) is a marker of HBV past exposure or current infection. In low prevalence populations, false positive results are possible.

HBeAg (early antigen) is a marker of infectivity and viral activity, whose presence indicates high infectivity and risk for liver injury.

Anti-HBs (antibody to surface antigen) is produced with recovery from infection, or in response to immunization. Over time, titer may decline to undetectable levels.

Anti-HBe is found in past/resolved infection. In most chronic carriers it indicates a less infectious state and a lower risk of liver injury.

Interpretation of diagnostic test results for HBV (HBsAg, anti-HBs, total anti-HBc, +/- anti-HBc IgM)

Primary tests		Optional tests		Interpretation
HBsAg	Anti-HBs	Anti-HBc	Anti-HBc IgM	
Negative	Negative	Negative	Not required	Not exposed and susceptible. Target for vaccination.
Negative	Positive	Negative	Not required	Already immune due to vaccination.
Negative	Positive	Positive	Not required	Immune due to previous infection.
Positive	Negative	Positive	Positive	Infected – acute infection or flare up of chronic
Positive	Negative	Positive	Negative	Infected – chronic infection
Negative	Negative	Positive	Negative	Four possible interpretations*

* Note: Very rarely an isolated anti-HBc total will be the only detectable marker. There are 4 possible interpretations for this finding:

- False positive result in low prevalence populations.
- Resolving acute infection before the appearance of anti-HBs
- Natural immunity with undetectable anti-HBs: due to test's lack of sensitivity and waning antibody titre over time
- May represent occult HBV infection (chronic infection with undetectable HBsAg): refer to specialist

Acute Infection

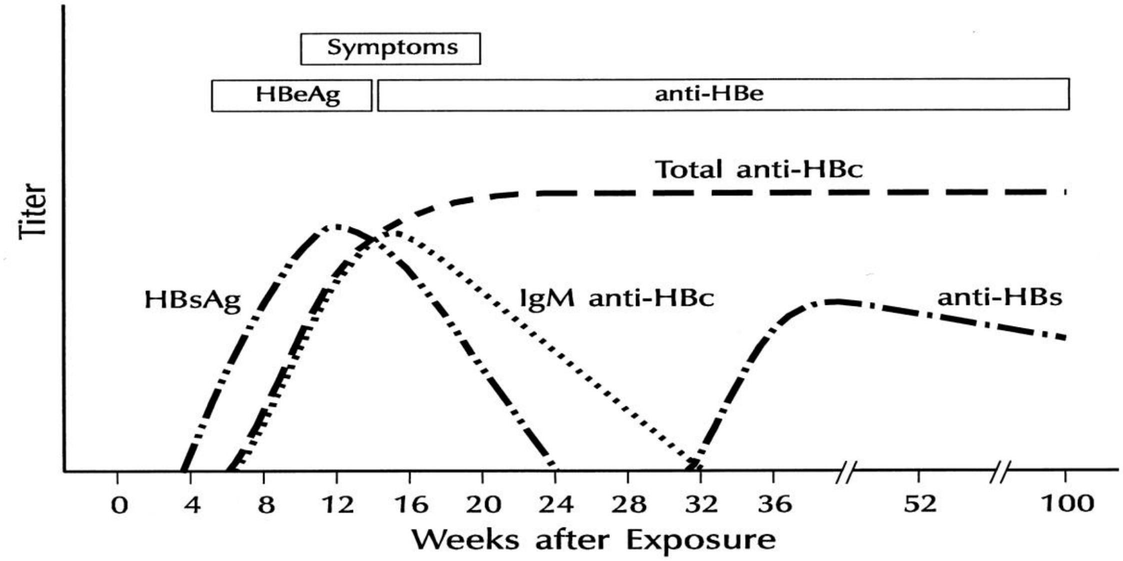

Chronic Infection

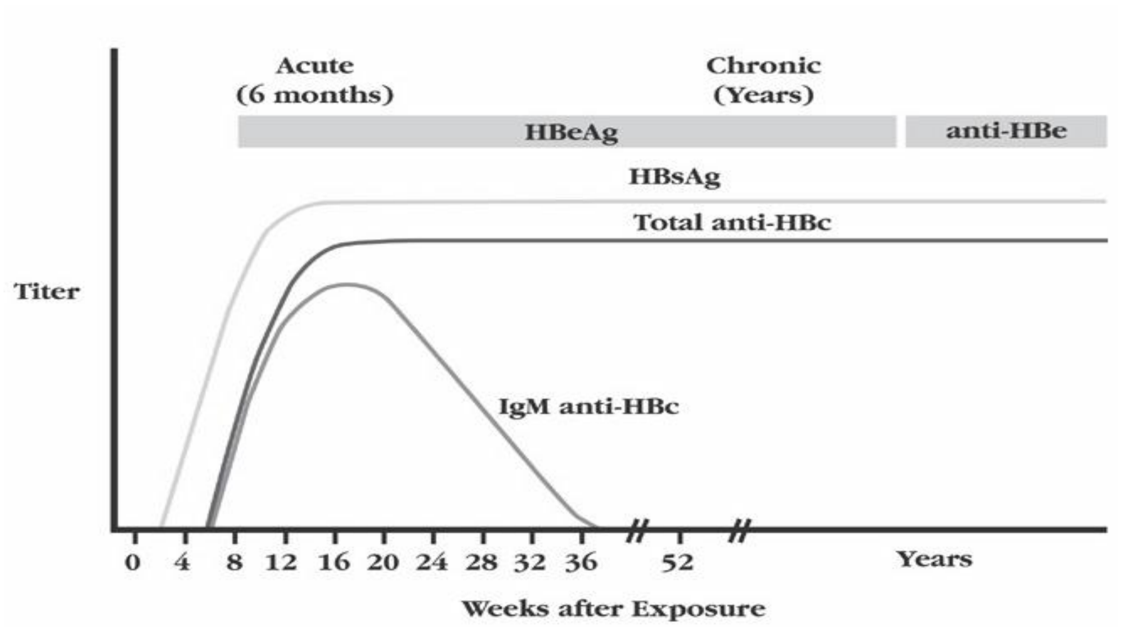

IMMIGRATION CLASS

3.6. Foreign Born

The term 'foreign born' applies to anyone born outside of their current country of permanent residence. It can apply to an immigrant, refugee or asylum seeker.

3.7. Immigrant

Immigrants enter another country, across national boundaries, for a permanent relocation. Most often immigrants must be employable to receive entry to countries such as Israel, the United States, Australia, New Zealand and Canada.

3.8. Refugee

A refugee is any person who owing to a well founded fear of being persecuted for reasons of race, religion, nationality, membership of a particular social group or political opinion, is outside the country of his/her nationality and is unable, or owing to such fear, is unwilling to avail himself/herself of the protection of that country.

3.9. Asylum Seeker

Asylum seekers are people who have applied for protection and are awaiting a determination of their status. Not all asylum seekers will be determined to be refugees.

STUDY TYPE

3.10. Cross-sectional study

A cross-sectional study is an analytical study in which disease and exposure status is measured simultaneously in a given population. For the purpose of this analysis, cross-sectional studies can be thought of as providing a "snapshot" of the data used to assess the seroprevalence of the immigrant population.

3.11. Cohort Study

A cohort study is an analytical study where individuals with differing exposures to a suspected factor are identified and then observed for the occurrence of certain health effects over some period, commonly years rather than weeks or months. Cohort studies can either be performed prospectively or retrospectively from historical records.

COUNTRY OF ORIGIN CLASSIFICATION

3.12. World Bank Regions of Origin

We classified immigrants and refugees to a region of origin according to the World Bank Regions. (see Appendix A for list)

4.0. METHODS

4.1. STUDY SELECTION CRITERIA

Eligibility of studies for inclusion will be assessed independently by two reviewers (CR and CG). Titles and abstracts of publications will first be screened using broad eligibility criteria. The full text of screened articles will then be subjected to the inclusion criteria described below. Studies not satisfying these criteria will be excluded.

4.2. Inclusion Criteria

1) Analytical studies (retrospective or prospective cohort and cross-sectional studies) reporting on outcomes of seroprevalence of HBsAg, anti-HBc, HBeAg and/or anti-HBs in a foreign-born population.
2) Focus of the study must be on the foreign-born population (including immigrants, refugees or asylum seekers) or a mixed population but with outcomes stratified by country of birth.
3) Studies that look at the seroprevalence of pregnant women or adopted children are also included.
4) The host country of the foreign-born population in the study must be Canada, United States, Japan, Australia, New Zealand, or a country in Western Europe, including Israel.
5) The study is written in English, French or Italian.

4.3. Exclusion Criteria

1) Case reports, conference abstracts, editorials, literature reviews, or reviews describing seroprevalence of HBsAg in foreign-born populations.
2) Studies that describe the seroprevalence of Hepatitis B markers in a population that is not representative of the overall immigrant or refugee population. For example, studies that represent sex workers, hospitalized immigrants, or immigrants who all have HIV or hepatocellular carcinoma, will be excluded.
3) Studies that do not report crude numbers to calculate seroprevalence or studies that report age-adjusted seroprevalence will be excluded.

5.0. SEARCH STRATEGY

5.1. Electronic Databases

Relevant studies were identified from a systematic review of 4 electronic databases: MEDLINE, MEDLINE In-Process, EMBASE, and the Cochrane Database of Systematic Reviews. Duplicate entries will be removed and all citations will be managed with EndNote x4.

5.2. Search Terms

The following search strategy was employed in every database searched:

1 exp Hepatitis B/

2 (hepatitis b or hepatitis b virus or chronic hepatitis b or hbv or chb).tw.

**3**	1 or 2
**4**	exp "Emigration and Immigration"/
**5**	(resettlement or re-settlement or border crossing or newcomer or naturalized citizen or nonnative or settler or new arrival or displaced person or in-migration or migration or migrant or immigrant or immigration or emigrant or emigration).tw.
**6**	4 or 5
**7**	exp Refugees/
**8**	(asylum seeker or refugee or displaced person or alien).tw.
**9**	7 or 8
**10**	3 and (6 or 9)

5.3 Hand Searches

Additional articles will be identified by reviewing the reference list of included articles in our study. These additional articles must satisfy the aforementioned inclusion criteria before becoming included articles.

5.4 Grey Literature

We will search the following organizations for any literature or documentation on the seroprevalence of infection or immunity in Immigrants and refugees: American Association for the Study of Liver Diseases (AASLD), Infectious Disease Society of America (IDSA), World Health Organization (WHO), Canadian Liver Foundation (CLF), American Society for Tropical Medicine and Hygiene (ASTMH), and the Canadian Association of Gastroenterology (CAG).

5.5. Quality Assessment

Since we will be examining seroprevalence studies, a non-observational epidemiological study design, we have approached the issue of quality assessment different from traditional systematic reviews. We deemed a seroprevalence study to be of good quality if the sample being screened is well representative of the general immigrant and refugee population within the host country, at the time the study took place. In our study exclusion criteria, we already excluded seroprevalence studies that examined HBsAg seroprevalence explicitly in immigrants and refugees who were not representative of the entire population, i.e. IV-drug users, sex workers, etc…

We will extract information on the participant selection method (i.e. clinic/hospital-based screening, immigration or refugee policy screening, screening of pregnant women, etc…) to ascertain if the population was by and large asymptomatic, and will examine our seroprevalence estimates in relation to this variable.

6.0. DATA EXTRACTION

The titles and abstracts of all identified studies from the search of the four electronic databases will be scanned by two reviewers (CR and CG) and classified as ‘not-relevant’

OR 'possibly relevant' using broad eligibility criteria. The full-text articles of those classified as 'possibly relevant' will be acquired and reviewed by the two reviewers and classified as 'included' or 'excluded' based upon the eligibility criteria and their ability to extract seroprevalence data from the study.

Data will be separately extracted by two readers (CR and LM) for all included articles. Data will be extracted in duplicate using a piloted data extraction form (Appendix 2). We will extract descriptive information from the articles about the age and sex composition of the immigrant population in the study, as well as the ethnic composition of the immigrant group(s) in the study. We will also ascertain the prevalence of any comorbidities in the population, such as HIV and Hepatitis C. We will obtain information on the seroprevalence of important HBV markers, such as HBsAg, anti-HBc, anti-HBs, and HBeAg. The seroprevalence of these markers will be stratified by the immigrant's region of origin, according to the World Bank classification, and by immigrant or refugee status. (see Data Extraction Form in Appendix B)

7.0. ANALYSES

Once the data has been extracted onto the data extraction forms, the results of the assessment of each included study will be entered into a Microsoft Access Database. The two readers who extracted the data will compare their results using the SAS proc compare command. Any disagreements will be resolved among the two readers, and if a suitable agreement cannot be met, a third reader (CG) will break the tie.

Our two primary outcomes are HBsAg seroprevalence and immunity. We will examine the seroprevalence of these two outcomes according to immigrant status and region of origin. We will run a random-effects meta-analysis to determine the pooled proportion to estimate the overall seroprevalence and its 95% confidence intervals. With recommendation from Dr. Guido Schwarzer, we will use a logit transformation to pool the proportions.

We will run a random-effects logistic regression model to examine the effect of region of origin, immigration status, and decade of publication on explaining chronic carriage and immunity. All statistical analysis will be done on R using the metaprop command developed by Guido Schwarzer.

8.0. REPORTING GUIDELINES

Study results will be reported according to PRISMA Guidelines for reporting systematic reviews and meta-analysis.

References

1. Custer B, Sullivan S, Hazlet T, Iloeje U, Veenstra D, Kowdley K. Global Epidemiology of Hepatitis B Virus. *J Clin Gastroenterol.* Nov-Dec 2004;38(3 supp):S158-168.

2. Kao J-H, Chen D-S. Global control of hepatitis B virus infection. *Lancet Infect Dis.* July 2002;2(7):395-403.

3. Greenaway C, Dongier P, Boivin J-F, Tapiero B, Miller M, Schwartzman K. Viral Hepatitis in Newly Arrived Immigrants and Refugees. Paper presented at: 56th Annual Meeting of the American Society of Tropical Medicine and Hygiene (ASTMH); November 4-8, 2007; Philadelphia, Pennsylvania.

4. Armstrong L, Goldstein S. Hepatitis B: Global epidemiology, diagnosis and prevention. In: Walker P, Barnett E, eds. *Immigrant Medicine.* Vol Section Four: Major Diseases and Disorders in Immigrants2007:321-341.

5. Plotkin S, Orenstein W. *Vaccines*. 4th Edition ed. Philadelphia: Saunders; 2004.

6. Sherman M. Surveillance for Hepatocellular Carcinoma and Early Diagnosis. *Clin Liver Dis.* 2007;11(4):817-837.

Appendix 1: World Bank Regions

East Asia and Pacific		
American Samoa	Marshall Islands	Singapore
Cambodia	Micronesia, Fe. Sts	Solomon Islands
China	Mongolia	Taiwan
Fiji	Myanmar	Thailand
Indonesia	Northern Mariana Islands	Timor-Leste
Japan	Pacific Islands	Tonga
Kiribati	Palau	Vanuatu
Korea, Dem. Rep.	Papua New Guinea	Vietnam
Lao PDR	Philippines	
Malaysia	Samoa	

Europe and Central Asia		
Albania	Kazakhstan	Romania
Armenia	Kosovo	Russian Federation
Azerbaijan	Kyrgyz Republic	Serbia
Belarus	Latvia	Slovak Republic
Bosnia and Herzegovina	Lithuania	Tajikistan
Bulgaria	Macedonia, FYR	Turkey
Croatia	Moldova	Turkmenistan
Georgia	Montenegro	Ukraine
Hungary	Poland	Uzbekistan

Latin America and the Caribbean		
Antigua and Barbuda	Dominican Republic	Panama
Argentina	Ecuador	Paraguay
Belize	El Salvador	Peru
Bolivia	Grenada	St. Kitts and Nevis
Brazil	Guatemala	St. Lucia
Central America	Guyana	St. Vincent and the Grenadines
Chile	Haiti	Suriname
Colombia	Honduras	Uruguay
Costa Rica	Jamaica	Venezuela, RB
Cuba	Mexico	
Dominica	Nicaragua	

Middle East and North Africa		
Algeria	Israel	Qatar
Bahrain	Jordan	Syrian Arab Republic
Djibouti	Lebanon	United Arab Emirates
Egypt, Arab Rep.	Libya	Tunisia
Iran, Islamic Rep.	Morocco	West Bank and Gaza

Iraq	Oman	Yemen, Rep.

**South Asia**		
Afghanistan	India	Pakistan
Bangladesh	Maldives	Sri Lanka
Bhutan	Nepal	

**Sub-Saharan Africa**		
Angola	Gabon	Niger
Benin	Gambia, The	Nigeria
Botswana	Ghana	Rwanda
Burkina Faso	Guinea	São Tomé and Principe
Burundi	Guinea-Bissau	Senegal
Cameroon	Kenya	Seychelles
Cape Verde	Lesotho	Sierra Leone
Central African Republic	Liberia	Somalia
Chad	Madagascar	South Africa
Comoros	Malawi	Sudan
Congo, De. Rep.	Mali	Swaziland
Congo, Rep.	Mauritania	Tanzania
Côte d'Ivoire	Mauritius	Togo
Equatorial Guinea	Mayotte	Uganda
Eritrea	Mozambique	Zambia
Ethiopia	Namibia	Zimbabwe

Appendix 2: Data Extraction Form

Seroprevalence Markers of Hepatitis B Viral Infection in Immigrant and Refugee Populations

Data Extraction Form

Part 1: COVERSHEET

1) Study number:

2) Data extracted by: ___________________

3) Date extraction completed: ______________
YYYY/MM/DD

4) Article title: ____

5) First author (Last Name): ______________________________________

6) Journal name: ____

7) Publication year: _________________

8) a) Author contacted ☐ Yes ☐ No
b) If Yes. Date contacted: ______________
YYYY/MM/DD
c) If Yes: Author e-mail: ______________________________________

9) a) Final status ☐ Included ☐ Excluded
b) Reason for exclusion: ____

10) Notes: ____
____
____
____

Part 2: STUDY POPULATION CHARACTERISTICS

11a) Type of publication:
- ☐ Peer-reviewed paper
- ☐ Unpublished report
- ☐ Other

11b) If "Other publication type" then other is: ________________

12a) Start date of study: ______________
YYYY/MM/DD

12b) End date of study: ______________
YYYY/MM/DD

13a) Country of Study: _________________________

13b) City (if applicable):________________________

14a) Study design:
- ☐ Ecologic
- ☐ Cross-sectional
- ☐ Case-control
- ☐ Prospective cohort
- ☐ Retrospective cohort
- ☐ Case-Series
- ☐ Other

14b) If "Other study design" then other is:_____________________________________

15a) What gender is being studied?
- ☐ Male
- ☐ Female
- ☐ Both
- ☐ Not mentioned

15b) What proportion of the study population is male? ______________________

15c) Are pregnant females included? ☐ Yes ☐ No ☐ Not specified

15d) If yes, then what proportion of females are pregnant? ______________________

16a) Exclusive category of Immigration status of study participants:

☐ Immigrant	☐ Refugee
☐ Asylum Seeker	☐ Foreign born
☐ Mixed	☐ Other
☐ Adopted children	☐ Not Mentioned

16b) If "Other exclusive category of immigration status" then other is:_____________________

16c) If immigration status is mixed then the included categories are:

c1) Immigrant ☐ Yes ☐ No
c2) Refugee ☐ Yes ☐ No
c3) Asylum Seeker ☐ Yes ☐ No
c4) Foreign born ☐ Yes ☐ No
c5) Other ☐ Yes ☐ No

16d) If "Other mixed category of immigration status" then other is:________________________

17a) Age (years) of the screened population:

a1) Mean ______________________________
a2) Median ____________________________
a3) Range low: _____________
a4) Range high: _____________

17b) Is the outcome data stratified by age? ☐ Yes ☐ No ☐ Not specified

18a) Is the outcome data stratified by country of origin? ☐ Yes ☐ No

18b) Exclusive Country of Origin:

☐ Not mentioned
☐ Mixed
☐ Latin America and Caribbean
☐ Eastern Europe and Central Asia
☐ Middle East & North Africa
☐ Sub-Saharan Africa
☐ South Asia
☐ East Asia & Pacific
☐ Non-World Bank Region _____________
☐ Other

18c) If "Other Exclusive Country of Origin", then other is:________________________

19a) If Mixed Country of Origin the regions of origin are included?

a1) Latin America and Caribbean ☐ Yes ☐ No
a2) Eastern Europe and Central Asia ☐ Yes ☐ No
a3) Middle East & North Africa ☐ Yes ☐ No
a4) Sub-Saharan Africa ☐ Yes ☐ No
a5) South Asia ☐ Yes ☐ No
a6) East Asia & Pacific ☐ Yes ☐ No
a7) Other/Unknown ☐ Yes ☐ No

19b) If "Other Mixed Country of Origin" then other is:__________________________________

20) Does the underlying population have co morbidities or confounders?

☐ Yes ☐ No ☐ Not specified

21a) If yes, what Comorbidities/Confounders are present?

a1)	Tuberculosis	☐ Yes	☐ No
a2)	Intestinal Parasites	☐ Yes	☐ No
a3)	Malaria	☐ Yes	☐ No
a4)	Other Viral Hepatitis Infections	☐ Yes	☐ No
a5)	Other	☐ Yes	☐ No

21b) If "Other Comorbidities/ Confounders" then other is:__________________________

Part 3: Risk of Bias/Quality Assessment

**A: SELECTION BIAS**		
22. How was the recruitment of study participants carried out?	☐ Clinic or Hospital Based Screening<br>☐ Screening Upon Arrival or at a Receiving Centre<br>☐ Pregnant Women Screening<br>☐ Invited for Screening<br>☐ Other	
23 What was the non-response rate or drop-out rate?		
**B: INFORMATION BIAS**		
24. What was the testing method?	☐ ELISA or EIA<br>☐ Reverse passive hemagglutination (RPHA)<br>☐ Radioimmunoassay (RIA)<br>☐ Other<br>☐ Not specified	
25. Was testing done the same way in the entire study population?	☐ Yes<br>☐ No<br>☐ Unable to tell	
**C: CONFOUDERS**		
26. List the major confounders (HIV status, IV Drug use, Homelessness, MSM) adjusted in the analysis or design (i.e. by matching)?	*Confounder*	Analysis or Match
**27. MOST IMPORTANT DESIGN FLAWS:**		

PART 4: SEROPREVALENCE DATA

A) Hepatitis B Surface Antigen (HBsAg)

28) Number of participants screened__________________
29) Number of participants positive __________________

30) HBsAg Total Seroprevalence (29/28):_____________________

IF Mixed Country of Origin has stratified outcomes:

31) Latin America and Caribbean ☐ Yes ☐ No
  - a1) Number of participants screened: Number _________________________
  - a2) Number of participants positive: Number _________________________
  - a3) HBsAg Seroprevalence: Number _________________________

32) Eastern Europe and Central Asia ☐ Yes ☐ No
  - a1) Number of participants screened: Number _________________________
  - a2) Number of participants positive: Number _________________________
  - a3) HBsAg Seroprevalence: Number _________________________

33) Middle East & North Africa ☐ Yes ☐ No
  - a1) Number of participants screened: Number _________________________
  - a2) Number of participants positive: Number _________________________
  - a3) HBsAg Seroprevalence: Number _________________________

34) Sub-Saharan Africa ☐ Yes ☐ No
  - a1) Number of participants screened: Number _________________________
  - a2) Number of participants positive: Number _________________________
  - a3) HBsAg Seroprevalence: Number _________________________

35) South Asia ☐ Yes ☐ No
  - a1) Number of participants screened: Number _________________________
  - a2) Number of participants positive: Number _________________________
  - a3) HBsAg Seroprevalence: Number _________________________

36) East Asia & Pacific ☐ Yes ☐ No
  - a1) Number of participants screened: Number _________________________
  - a2) Number of participants positive: Number _________________________
  - a3) HBsAg Seroprevalence: Number _________________________

37) Combined Africa (Non-WB) ☐ Yes ☐ No
  - a1) Number of participants screened: Number _________________________
  - a2) Number of participants positive: Number _________________________
  - a3) HBsAg Seroprevalence: Number _________________________

38) Combined Asia (Non-WB) ☐ Yes ☐ No

a1) Number of participants screened: Number ________________________
a2) Number of participants positive: Number ________________________
a3) HBsAg Seroprevalence: Number ________________________

B) Immunity

39a) Study reports the seroprevalence of immunity ☐ Yes ☐ No
Defined as either the presence of Anti-HBs or the presence of both Anti-HBs and Anti-HBc

39b) Which type of immunity is being reported ☐ Anti-HBs alone (vaccinated)
☐ Anti-HBs and Anti-HBc (Resolved infection)
☐ Any immunity
☐ Not specified

40) Number of participants screened__________________
41) Number of participants immune __________________

42) Total Seroprevalence of immunity (41/40):_____________________

IF Mixed Country of Origin has stratified outcomes:

43) Latin America and Caribbean ☐ Yes ☐ No
a1) Number of participants screened: Number ________________________
a2) Number of participants positive: Number ________________________
a3) Seroprevalence of immunity: Number ________________________

44) Eastern Europe and Central Asia ☐ Yes ☐ No
a1) Number of participants screened: Number ________________________
a2) Number of participants positive: Number ________________________
a3) Seroprevalence of immunity: Number ________________________

45) Middle East & North Africa ☐ Yes ☐ No
a1) Number of participants screened: Number ________________________
a2) Number of participants positive: Number ________________________
a3) Seroprevalence of immunity: Number ________________________

46) Sub-Saharan Africa ☐ Yes ☐ No
a1) Number of participants screened: Number ________________________
a2) Number of participants positive: Number ________________________
a3) Seroprevalence of immunity: Number ________________________

47) South Asia ☐ Yes ☐ No
a1) Number of participants screened: Number ________________________
a2) Number of participants positive: Number ________________________
a3) Seroprevalence of immunity: Number ________________________

48) East Asia & Pacific ☐ Yes ☐ No
- a1) Number of participants screened: Number ___________________________
- a2) Number of participants positive: Number ___________________________
- a3) Seroprevalence of immunity: Number ___________________________

49) Combined Africa (Non-WB) ☐ Yes ☐ No
- a1) Number of participants screened: Number ___________________________
- a2) Number of participants positive: Number ___________________________
- a3) Seroprevalence of immunity: Number ___________________________

50) Combined Asia (Non-WB) ☐ Yes ☐ No
- a1) Number of participants screened: Number ___________________________
- a2) Number of participants positive: Number ___________________________
- a3) Seroprevalence of immunity: Number ___________________________

<u>C) Antibody to Hepatitis B Surface Antigen alone (anti-HBs)</u>

51) Study reports seroprevalence of anti-HBs ☐ Yes ☐ No
Defined as the presence of anti-HBs **alone**. This informs us if the subject was vaccinated or not.

52) Number of participants screened____________________
53) Number of participants positive ____________________

54) anti-HBs (alone) Total Seroprevalence (53/52):_______________________

IF <u>Mixed</u> Country of Origin has stratified outcomes:

55) Latin America and Caribbean ☐ Yes ☐ No
- a1) Number of participants screened: Number ___________________________
- a2) Number of participants positive: Number ___________________________
- a3) anti-HBs Seroprevalence: Number ___________________________

56) Eastern Europe and Central Asia ☐ Yes ☐ No
- a1) Number of participants screened: Number ___________________________
- a2) Number of participants positive: Number ___________________________
- a3) anti-HBs Seroprevalence: Number ___________________________

57) Middle East & North Africa ☐ Yes ☐ No
- a1) Number of participants screened: Number ___________________________
- a2) Number of participants positive: Number ___________________________
- a3) anti-HBs Seroprevalence: Number ___________________________

58) Sub-Saharan Africa ☐ Yes ☐ No
- a1) Number of participants screened: Number ___________________________

a2) Number of participants positive: Number ___________________________
a3) anti-HBs Seroprevalence: Number ___________________________

59) South Asia ☐ Yes ☐ No
a1) Number of participants screened: Number ___________________________
a2) Number of participants positive: Number ___________________________
a3) anti-HBs Seroprevalence: Number ___________________________

60) East Asia & Pacific ☐ Yes ☐ No
a1) Number of participants screened: Number ___________________________
a2) Number of participants positive: Number ___________________________
a3) anti-HBs Seroprevalence: Number ___________________________

61) Combined Africa (Non-WB) ☐ Yes ☐ No
a1) Number of participants screened: Number ___________________________
a2) Number of participants positive: Number ___________________________
a3) anti-HBs Seroprevalence: Number ___________________________

62) Combined Asia (Non-WB) ☐ Yes ☐ No
a1) Number of participants screened: Number ___________________________
a2) Number of participants positive: Number ___________________________
a3) anti-HBs Seroprevalence: Number ___________________________

<u>Other References</u> (List number of citation and first author of potentially interesting follow-up articles):

____
____
____
____
____
