# Supplementary material for: Seroprevalence of Chronic Hepatitis B Virus Infection and Prior Immunity in Immigrants and Refugees: A Systematic Review and Meta-Analysis
Source: PLoS One. 2012 Sep 5;7(9):e44611. doi: 10.1371/journal.pone.0044611 (PMC3434171; doi:10.1371/journal.pone.0044611)
Supplement: Text S2 — Supplementary tables. (DOCX) [file pone.0044611.s005.docx]

## Text S2: Supplemental Material for Manuscript

Appendix Table 1: Foreign-born population by region of origin in major immigrant-receiving countries

|  | Report Year | Latin America and Caribbean | Eastern Europe and Central Asia | Middle East and North Africa | Sub-Saharan Africa | South Asia | East Asia and the Pacific | Total |
| --- | --- | --- | --- | --- | --- | --- | --- | --- |
| **North America** |  |  |  |  |  |  |  |  |
| Canada | 2006[1] | 698,935 | 712,825 | 413,450 | 252,645 | 755,445 | 1,438,045 | **4,271,345** |
| United States | 2009[2] | 20,455,547 | 2,944,186 | 893,333 | 1,492,785 | 2,713,675 | 7,000,964 | **35,500,490** |
| **Europe** |  |  |  |  |  |  |  |  |
| Austria | 2010[3] | 18,017 | 839,271 | 45,691 | 21,222 | 26,972 | 41,955 | **993,128** |
| Belgium | 2011[4] | 16,061 | 189,294 | 109,964 | 60,163 | 15,913 | 19,789 | **411,184** |
| Czech Republic | 2010[5] | 1,793 | 304,057 | 4,843 | 2,028 | 2,037 | 75,356 | **390,114** |
| Denmark | 2009[6] | 8,886 | 123,509 | 59,549 | 23,315 | 33,916 | 36,762 | **285,937** |
| Finland | 2011[7] | 4,833 | 106,488 | 17,486 | 18,601 | 11,084 | 22,621 | **181,113** |
| France | 2006[8] | 166,961 | 421,999 | 1,131,726 | 425,009 | 60,329 | 141,709 | **2,347,733** |
| Germany | 2010[9] | 104,193 | 3,706,019 | 330,191 | 154,627 | 163,751 | 325,441 | **4,784,222** |
| Greece | 2001[10] | 859 | 621,945 | 23,708 | 4,377 | 24,392 | 8,595 | **683,876** |
| Israel | 2008[11] | 65,940 | 555,640 | 398,480 | 91,000 | 20,710 | 15,850 | **1,147,620** |
| Italy | 2010[12] | 229,092 | 2,014,922 | 642,720 | 165,645 | 320,030 | 311,936 | **3,684,345** |
| Netherlands | 2011[13] | 319,995 | 407,683 | 274,286 | 114,479 | 67,195 | 211,347 | **1,394,985** |
| Norway | 2011[14] | 23,628 | 156,668 | 51,018 | 50,398 | 47,067 | 65,795 | **394,574** |
| Portugal | 2010[15] | 125,161 | 123,261 | 3,040 | 106,161 | 9,700 | 17,953 | **385,276** |
| Republic of Ireland | 2006[16] | 7,951 | 143,287 | 7,789 | 38,547 | 18,129 | 31,172 | **246,875** |
| Spain | 2007[17] | 1,569,837 | 896,697 | 642,240 | 173,949 | 69,881 | 134,138 | **3,486,742** |
| Sweden | 2011[18] | 74,709 | 377,986 | 261,144 | 95,757 | 55,601 | 100,373 | **965,570** |
| Switzerland | 2010[19] | 48,501 | 455,968 | 40,642 | 52,818 | 51,115 | 42,179 | **691,223** |
| United Kingdom | 2001[20] | 339,979 | 293,989 | 218,186 | 759,137 | 1,030,483 | 359,990 | **3,001,764** |
| **Oceania** |  |  |  |  |  |  |  |  |
| Australia | 2006[21] | 86,385 | 374,825 | 193,216 | 210,934 | 259,662 | 1,016,118 | **2,141,140** |
| New Zealand | 2006[22] | 7,638 | 22,422 | 16,533 | 59,118 | 56,391 | 329,289 | **491,391** |

# Appendix Table 2: 110 included studies in systematic review

| Source | Year of Study | Country of Landing | Region of Origin | Age range, y | Immigrant Status | Population Screened | HBsAg Seroprevalence (%) | anti-HBs Seroprevalence (%) |
| --- | --- | --- | --- | --- | --- | --- | --- | --- |
| Adair et al, 1999[23] | 1997 | United States | Sub-Saharan |  | Immigrant | 73 | 13.7 |  |
| Almog et al, 1999[24] | 1992-1993 | Israel | Eastern Europe | 17-49 | Immigrant | 599 | 4.8 | 19.2 |
| Arevalo et al, 1989[25] | 1983-1987 | United States | Mixed | (Median: 26) | Immigrant | 277 | 8.3 |  |
| Aubert et al, 2010[26] | 2007-2008 | France | Mixed | 18-88 (Mean: 40) | Immigrant | 481 | 9.4 | 64.7 |
| Aweis et al, 2001[27] | 2000 | United Kingdom | Sub-Saharan | 1-80 | Immigrant | 317 | 7.3 |  |
| Baldo et al, 2000[28] | 1996 | Italy | Mixed | 18-43 (Mean: 28) | Immigrant | 255 | 3.1 |  |
| Barry et al, 1983[29] | 1979-1980 | United States | East Asia & Pacific |  | Refugee | 83 | 15.7 | 56.6 |
| Beggio et al, 2007[30] |  | Italy | Mixed |  | Immigrant | 199 | 4.0 | 32.2 |
| Ben-Porath et al, 1986[31] |  | Israel | Sub-Saharan |  | Refugee | 357 | 12.6 | 44.8 |
| Bjerke et al, 2011[32] | 2009 | Norway | South Asia | 18-44 (Mean: 27.3) | Immigrant | 206 | 0.5 | 7.8 |
| Bonura et al, 2005[33] | 2001-2003 | Italy | Mixed | 18-44 (Mean: 30) | Immigrant | 310 | 4.2 |  |
| Bottecchia et al, 2011[34] | 2007-2008 | Spain | Mixed | (Median: 51.5) | Immigrant | 1,718 | 7.0 |  |
| Caruana et al, 2005[35] |  | Australia | East Asia & Pacific | 15-92 (Median: 44) | Mixed | 327 | 8.6 | 76.5 |
| Catanzaro et al, 1982[36] | 1980-1981 | United States | East Asia &Pacific |  | Refugee | 301 | 14.0 |  |
| CDC, 2006[37] | 2005 | United States | Mixed | 20-83 (Median: 45) | Immigrant | 915 | 11.7 |  |
| CDC, 1991[38] | 1979-1991 | United States | Mixed |  | Refugee | 36,812 | 15.0 |  |
| Chadwick et al, 1982[39] |  | United Kingdom | East Asia & Pacific |  | Refugee | 632 | 15.0 |  |
| Chaudhary et al, 1981[40] | 1979-1980 | Canada | East Asia & Pacific |  | Refugee | 14,347 | 11.6 | 48.9 |
| Chaves et al, 2009[41] | 2004-2008 | Australia | East Asia & Pacific | 16-86 (Median: 30) | Refugee | 141 | 14.2 |  |
| Chemtob et al, 1991[42] | 1987 | Israel | Sub-Saharan | 2-67 (Mean: 23) | Refugee | 144 | 18.8 | 41.7 |
| Chiaramonte et al, 1998[43] | 1989-1995 | Italy | Mixed | 0-76 (Mean: 28.5) | Immigrant | 1,683 | 8.9 |  |
| Chironna et al, 2003[44] | 2000 | Italy | Mixed | 1-55 (Mean: 24) | Refugee | 1,005 | 3.9 |  |
| Chironna et al, 2000[45] |  | Italy | Eastern Europe | 2-72 (Mean: 25) | Refugee | 670 | 13.6 | 47.6 |
| Chironna et al, 2001[46] | 1999 | Italy | Eastern Europe | 2-72 (Mean: 13) | Refugee | 526 | 2.9 | 12.0 |
| Christenson et al, 1997[47] |  | Sweden | Mixed |  | Immigrant | 133 | 1.5 |  |
| Dalekos et al, 1995[48] |  | Greece | Eastern Europe | 0-81 | Refugee | 1,025 | 22.1 | 40.5 |
| Denburg et al, 2007[49] | 2006 | Canada | East Asia & Pacific |  | Refugee | 64 | 14.1 | 43.8 |
| Denis et al, 1994[50] | 1992-1993 | France | Mixed |  | Immigrant | 5,125 | 2.5 |  |
| Elefsiniotis et al, 2007[51] | 2003-2005 | Greece | Mixed | 16-45 | Immigrant | 8,698 | 3.5 |  |
| Engebretsen et al, 1984[52] | 1981-1983 | United States | East Asia &Pacific |  | Refugee | 552 | 11.8 |  |
| Entzel et al, 2003[53] | 1999-2000 | United States | Latin America | 0-6 (Mean: 3.5) | Refugee (Children) | 244 | 0.4 |  |
| Fabris et al, 2008[54] |  | Italy | Mixed | (Mean: 27.7) | Immigrant | 47 | 6.4 |  |
| Faustini et al, 1994[55] | 1991 | Italy | Mixed | 1-67 (Mean: 24) | Immigrant | 138 | 5.1 | 27.8 |
| Fitzpatrick et al, 1987[56] | 1981-1985 | United States | East Asia & Pacific | 11-19 (Mean: 14.7) | Refugee (Children) | 74 | 13.5 |  |
| Flatau et al, 1993[57] | 1991 | Israel | Sub-Saharan | 1-76 (Mean: 29) | Refugee | 200 | 11.5 | 47.1 |
| Franco-Paredes et al, 2007[58] | 2005-2006 | United States | Sub-Saharan | (Mean: 25) | Refugee | 31 | 32.3 |  |
| Friedman et al, 1998[59] | 1984-1985 | United States | Mixed | 14-44 (Mean: 26) | Immigrant | 288 | 5.9 |  |
| Garcia-Samaniego et al, 1994[60] | 1992-1993 | Spain | Sub-Saharan | 16-53 (Mean: 31.4) | Immigrant | 435 | 20.7 |  |
| Germinario et al, 2000[61] |  | Italy | Eastern Europe | 0-10 | Refugee (Children) | 415 | 0.5 |  |
| Gish et al, 2011[62] |  | United States | East Asia & Pacific |  | Immigrant | 1,798 | 6.0 | 54.0 |
| Gjerdingen et al, 1997[63] | 1994-1995 | United States | East Asia & Pacific | (Mean: 27.7) | Refugee | 429 | 17.9 | 42.2 |
| Gjorup et al, 2003[64] | 1994-1995 | Denmark | Mixed | 5-19 | Immigrant (Children) | 144 | 2.1 |  |
| Glikberg et al, 1997[65] |  | Israel | Eastern Europe |  | Immigrant | 102 | 15.7 |  |
| Goldenring et al, 1983[66] | 1979-1982 | United States | East Asia & Pacific | (Mean: 19.5) | Immigrant (Children) | 89 | 16.9 |  |
| Goodman et al, 1984[67] | 1980-1981 | United States | East Asia & Pacific |  | Refugee | 849 | 11.7 | 55.5 |
| Hayes et al, 1998[68] | 1994 | United States | Mixed |  | Refugee | 124 | 4.0 | 21.1 |
| Hill et al, 1991[69] |  | United States | East Asia & Pacific |  | Refugee | 679 | 17.4 |  |
| Hornstein et al, 1991[70] | 1985-1988 | Israel | Sub-Saharan |  | Refugee | 224 | 16.5 | 47.2 |
| Huerga et al, 2002[71] | 1989-2001 | Spain | Sub-Saharan | 0-13 (Mean: 7) | Immigrant (Children) | 75 | 6.7 | 32.0 |
| Hurie et al, 1995[72] | 1990-1993 | United States | Eastern Europe |  | Refugee | 496 | 0.4 |  |
| Hurie et al, 1992[73] | 1984-1989 | United States | East Asia & Pacific | 0-75 (Mean: 22.6) | Refugee | 754 | 18.7 | 45.1 |
| Jenista et al, 1987[74] |  | United States | Mixed |  | Adopted Children | 68 | 5.9 |  |
| Jensen et al, 2003[75] | 2000-2001 | Denmark | Mixed |  | Immigrant | 1,010 | 1.7 |  |
| Judson et al, 1984[76] | 1981-1982 | United States | East Asia & Pacific |  | Refugee | 516 | 14.7 |  |
| King et al, 2001[77] | 2000-2001 | Australia | Mixed |  | Asylum Seeker | 7,000 | 2.5 |  |
| Kulstrunk et al, 1992[78] |  | Switzerland | Eastern Europe | 0-53 | Refugee | 1,299 | 9.1 | 46.8 |
| Lange et al, 1987[79] | 1985-1986 | United States | East Asia & Pacific | 0.2-12 | Adopted Children | 360 | 2.8 | 3.4 |
| Levinne et al, 1980[80] | 1979 | Canada | East Asia & Pacific |  | Refugee | 192 | 10.9 |  |
| Levy et al, 2010[81] |  | United States | Mixed | (Median: 24) | Immigrant | 701 | 0.6 |  |
| Lifson et al, 2002[82] | 1999 | United States | Mixed | (Mean: 23) | Refugee | 2,353 | 7.4 | 36.7 |
| Lin et al, 2007[83] | 2001-2006 | United States | East Asia & Pacific | 18-101 (Median: 53) | Immigrant | 2,386 | 10.7 |  |
| Lopez-Velez et al, 2003[84] | 1989-1999 | Spain | Mixed | 0-82 (Median: 28) | Mixed | 662 | 7.1 |  |
| Majori et al, 2008[85] | 2004-2005 | Italy | Sub-Saharan | 0-60 (Mean: 31.7) | Illegal Immigrant | 182 | 9.3 | 39.6 |
| Malamitsi-Puchner et al, 1996[86] |  | Greece | Eastern Europe | 14-42 (Mean: 25.1) | Refugee | 500 | 13.4 | 53.0 |
| Manzardo et al, 2008[87] | 2001-2004 | Spain | Mixed | 0-80 (Mean: 29.5) | Mixed | 1,905 | 7.7 |  |
| Martin et al, 2006[88] | 2003-2004 | Australia | Mixed | 0-89 | Refugee | 1,974 | 5.4 |  |
| Meints et al, 2010[89] | 2003-2006 | United States | Mixed |  | Immigrant | 1,685 | 3.1 |  |
| Meropol, 1995[90] | 1991-1993 | United States | Mixed | 1-18 (Median: 8.2) | Refugee (Children) | 81 | 7.4 | 43.2 |
| Milionis, 2010[91] | 2000-2009 | Greece | Eastern Europe | 10-23 (Mean: 15.6) | Immigrant (Children) | 504 | 11.7 | 35.1 |
| Museru et al, 2010[92] | 2003-2007 | United States | Mixed |  | Refugee | 6,347 | 10.7 |  |
| Museru et al, 2009[93] | 2005-2008 | United States | Sub-Saharan | 16-48 (Mean: 28) | Refugee | 74 | 23.0 |  |
| Nahmias et al, 1993[94] | 1978-1991 | Israel | Sub-Saharan |  | Refugee | 6,230 | 11.5 |  |
| Nelson et al, 1997[95] | 1994-1995 | United States | East Asia & Pacific | 19-71 (Median: 34) | Immigrant | 96 | 13.5 | 49.0 |
| Ooi et al, 2006[96] | 1999-2002 | United States | Mixed | (Mean: 39) | Immigrant | 209 | 1.9 | 20.0 |
| Palumbo et al, 2007(a)[97] | 2005 | Italy | Mixed | 15-47 (Mean: 22.6) | Refugee | 556 | 10.8 |  |
| Palumbo et al, 2007(b)[98] | 2005-2006 | Italy | Latin America | 15-43 (Mean: 21.3) | Refugee | 130 | 10.8 |  |
| Palumbo et al, 2008[99] | 2003-2004 | Italy | Mixed | 15-39 (Mean: 24) | Refugee | 890 | 9.3 |  |
| Panagopoulouset al, 2004[100] | 1994-2002 | Greece | Mixed |  | Immigrant | 3,017 | 4.7 |  |
| Papaevangelou et al, 2006[101] | 2003 | Greece | Mixed |  | Immigrant | 596 | 8.2 |  |
| Parenti et al, 1987[102] | 1980-1984 | United States | Sub-Saharan | 0-55 | Refugee | 53 | 9.4 |  |
| Patel et al, 2002[103] | 1991-1999 | United States | East Asia & Pacific |  | Immigrant | 743 | 13.9 |  |
| Perez-Molina et al, 2011[104] | 1989-2008 | Spain | Mixed | (Mean: 31.5) | Immigrant | 322 | 10.6 |  |
| Pottie et al, 2007[105] | 2004-2005 | Canada | Mixed |  | Refugee | 112 | 5.4 |  |
| Ranger et al, 1990[106] | 1984-1988 | France | Mixed | 13-47 (Mean: 28) | Immigrant | 1,206 | 2.6 |  |
| Rein et al, 2010[107] | 2006-2008 | United States | Mixed |  | Refugee | 31,980 | 2.8 |  |
| Roberts et al, 1985[108] | 1980-1982 | United States | East Asia & Pacific | 16-51 (Mean: 25.3) | Refugee | 97 | 5.2 |  |
| Roudot-Thoraval et al, 1989[109] |  | France | Mixed | 16-44 (Mean: 26) | Immigrant | 628 | 3.0 |  |
| Roussos et al, 2003[110] |  | Greece | Mixed | 18-69 (Mean: 31.7) | Refugee | 130 | 15.4 |  |
| Saiman et al, 2001[111] | 1997-1998 | United States | Mixed | 0-12 (Mean: 1.6) | Adopted Children | 499 | 2.8 | 35.1 |
| Salleras et al, 2009[112] | 2004 | Spain | Mixed |  | Immigrant | 114 | 0.9 |  |
| Sandler et al, 1977[113] |  | Israel | Middle East |  | Immigrant | 414 | 1.2 | 28.9 |
| Santantonio et al, 1993[114] | 1991 | Italy | Eastern Europe | 1-46 (Mean: 20) | Refugee | 393 | 19.1 | 37.2 |
| Sheikh et al, 2009[115] | 2005-2006 | Australia | Mixed | 1-17 | Refugee (Children) | 218 | 3.2 |  |
| Skinhoj et al, 1981[116] | 1979 | Denmark | East Asia &Pacific |  | Refugee | 564 | 10.5 |  |
| Skinhoj et al, 1983[117] | 1979-1980 | Denmark | East Asia & Pacific |  | Refugee | 533 | 10.3 |  |
| Skliros et al, 2001[118] |  | Greece | Eastern Europe | (Mean: 31.6) | Refugee | 154 | 7.1 |  |
| Skliros et al, 1999[119] |  | Greece | Eastern Europe | 1-75 | Refugee | 188 | 9.0 |  |
| Smith et al, 2000[120] | 1999 | Ireland | Eastern Europe |  | Refugee | 919 | 2.8 |  |
| Smith et al, 1984[121] |  | Australia | East Asia & Pacific |  | Refugee | 108 | 13.0 | 46.3 |
| Smith-Garcia et al, 1989[122] | 1978-1987 | United States | South Asia | 0-20 | Adopted Children | 76 | 6.6 |  |
| Stadler et al, 2008[123] | 1999-2006 | United States | Mixed | 0-16 (Mean: 2.2) | Adopted Children | 1,228 | 1.1 | 67.2 |
| Stroffolini et al, 2003[124] |  | Italy | Mixed |  | Immigrant | 597 | 5.9 |  |
| Tafuri et al, 2010[125] | 2008 | Italy | Mixed | 7-52 (Mean: 23.9) | Refugee | 529 | 8.3 |  |
| Tiong et al, 2006[126] | 2005-2006 | Australia | Sub-Saharan |  | Refugee | 184 | 8.2 |  |
| Tong et al, 1984[127] | 1982 | United States | East Asia & Pacific | (Mean: 52) | Immigrant | 243 | 13.6 | 56.4 |
| Toro et al, 2006[128] | 2002-2003 | Spain | Mixed | 0-89 (Median: 29.5) | Immigrant | 486 | 5.3 |  |
| Ugwu et al, 2008[129] | 1998-2001 | United States | Mixed | (Median: 19) | Refugee | 8,754 | 7.1 | 31.1 |
| van Steenbergen et al, 2001[130] | 1993-1998 | Netherlands | Mixed |  | Immigrant | 30,937 | 2.2 |  |
| Veldhuijzen et al, 2008[131] | 2004 | Netherlands | Mixed | 18-65 | Immigrant | 205 | 1.0 |  |
| Viviano et al, 2006[132] | 2002-2005 | Italy | Eastern Europe | 1-12 (Mean: 6.3) | Adopted Children | 75 | 4.0 | 41.3 |

Appendix Table 3: Characteristics of included studies reporting chronic hepatitis B infection

|  | **Number of Studies (%)** | **Total Sample Size (%)** |
| --- | --- | --- |
| Total | 110 | 209,822 |
|  |  |  |
| Immigrant Status |  |  |
| Immigrants | 44 (40.0) | 70,204 (33.5) |
| Adopted Children | 6 (5.5) | 2,306 (1.1) |
| Refugees | 56 (50.9) | 127,418 (60.7) |
| Asylum Seekers | 1 (0.9) | 7,000 (3.3) |
| Mixed | 3 (2.7) | 2,894 (1.4) |
|  |  |  |
| Age Group |  |  |
| Immigrants |  |  |
| Immigrant Adults | 25 (22.7) | 22,849 (10.9) |
| Immigrant Children | 4 (3.6) | 812 (0.4) |
| Adopted Children | 6 (5.5) | 2,306 (1.1) |
| No Age Reported | 15 (13.6) | 46,543 (22.2) |
|  |  |  |
| Refugees and Asylum Seekers |  |  |
| Refugee Adults | 27 (24.5) | 23,181 (11.0) |
| Refugee Children | 5 (4.5) | 1,032 (0.5) |
| No Age Reported | 25 (22.7) | 110,205 (52.5) |
|  |  |  |
| Mixed Adults | 3 (2.7) | 2,894 (1.4) |
|  |  |  |
| Exclusive Region of Origin |  |  |
| Latin America | 2 (1.8) | 374 (0.2) |
| Eastern Europe | 15 (13.6) | 7,865 (3.7) |
| Middle East & North Africa | 1 (0.9) | 414 (0.2) |
| Sub-Saharan Africa | 14 (12.7) | 8,579 (4.1) |
| South Asia | 2 (1.8) | 282 (0.1) |
| East Asia & The Pacific | 26 (23.6) | 26,957 (12.8) |
| Mixed | 50 (45.5) | 165,351 (78.8) |
|  |  |  |
| Country of Landing |  |  |
| Australia | 7 (6.4) | 9,952 (4.7) |
| Canada | 4 (3.6) | 14,715 (7.0) |
| Denmark | 4 (3.6) | 2,251 (1.1) |
| France | 4 (3.6) | 7,440 (3.5) |
| Greece | 9 (8.2) | 14,812 (7.1) |
| Ireland | 1 (0.9) | 919 (0.4) |
| Israel | 8 (7.3) | 8,270 (3.9) |
| Italy | 18 (16.3) | 8,600 (4.1) |
| Netherlands | 2 (1.8) | 31,142 (14.8) |
| Norway | 1 (0.9) | 206 (0.1) |
| Spain | 8 (7.3) | 5,717 (2.7) |
| Sweden | 1 (0.9) | 133 (0.1) |
| Switzerland | 1 (0.9) | 1,299 (0.6) |
| United Kingdom | 2 (1.8) | 949 (0.5) |
| United States | 40 (36.4) | 103,417 (49.3) |
|  |  |  |
| Participant Selection Method |  |  |
| Clinic/Hospital-Based Screening | 34 (30.9) | 13,704 (6.5) |
| Screening Upon Reception/Arrival | 42 (38.2) | 131,856 (62.8) |
| Pregnant Women | 18 (16.4) | 55,546 (26.5) |
| Invited for Screening | 8 (7.3) | 6,329 (3.0) |
| Other | 8 (7.3) | 2,387 (1.1) |
|  |  |  |
| Decade of Publication |  |  |
| Before 1980 | 1 (0.9) | 414 (0.2) |
| 1980-1989 | 23 (20.9) | 21,287 (10.1) |
| 1990-1999 | 26 (23.6) | 59,168 (28.2) |
| 2000-present | 60 (54.5) | 129,013 (61.5) |

Appendix Table 4: Characteristics of included studies reporting hepatitis B immunity

|  | **Number of Studies (%)** | **Total Sample Size (%)** |
| --- | --- | --- |
| Total | 39 | 40 330 |
|  |  |  |
| Immigrant Status |  |  |
| Immigrants | 12 (30.8) | 5,318 (13.2) |
| Adopted Children | 4 (10.3) | 1,889 (4.7) |
| Refugees | 22 (56.4) | 32,796 (81.3) |
| Asylum Seekers | 0 | 0 |
| Mixed | 1 (2.6) | 327 (0.8) |
|  |  |  |
| Age Group |  |  |
| Immigrants |  |  |
| Immigrant Adults | 7 (17.9) | 2,621 (6.5) |
| Immigrant Children | 2 (5.1) | 579 (1.4) |
| Adopted Children | 4 (10.3) | 1,889 (4.7) |
| No Age Reported | 3 (7.7) | 2,118 (5.3) |
|  |  |  |
| Refugees and Asylum Seekers |  |  |
| Refugee Adults | 13 (33.3) | 16,555 (41.0) |
| Refugee Children | 1 (2.6) | 81 (0.2) |
| No Age Reported | 8 (20.5) | 16,160 (40.0) |
|  |  |  |
| Mixed Adults | 1 (2.6) | 327 (0.8) |
|  |  |  |
| Exclusive Region of Origin |  |  |
| Latin America | 0 | 0 |
| Eastern Europe | 9 (23.1) | 6,164 (15.3) |
| Middle East & North Africa | 1 (2.6) | 121 (0.3) |
| Sub-Saharan Africa | 6 (15.4) | 1,055 (2.6) |
| South Asia | 1 (2.6) | 206 (0.5) |
| East Asia & The Pacific | 12 (30.8) | 19,146 (47.5) |
| Mixed | 10 (25.6) | 13,638 (33.8) |
|  |  |  |
| Country of Landing |  |  |
| Australia | 2 (5.1) | 435 (1.1) |
| Canada | 2 (5.1) | 14,411 (35.7) |
| Denmark | 0 | 0 |
| France | 1 (2.6) | 481 (1.2) |
| Greece | 3 (7.7) | 2,025 (5.0) |
| Ireland | 0 | 0 |
| Israel | 6 (15.4) | 2,095 (5.2) |
| Italy | 7 (17.9) | 2,254 (5.6) |
| Netherlands | 0 | 0 |
| Norway | 1 (2.6) | 206 (0.5) |
| Spain | 1 (2.6) | 75 (0.2) |
| Sweden | 0 | 0 |
| Switzerland | 1 (2.6) | 1,299 (3.2) |
| United Kingdom | 0 | 0 |
| United States | 15 (38.5) | 17,049 (42.3) |
|  |  |  |
| Participant Selection Method |  |  |
| Clinic/Hospital-Based Screening | 11 (28.2) | 3,646 (9.0) |
| Screening Upon Reception/Arrival | 18 (46.2) | 31,797 (78.8) |
| Pregnant Women | 2 (5.1) | 706 (1.8) |
| Invited for Screening | 4 (10.3) | 2,577 (6.4) |
| Other | 4 (10.3) | 1,604 (4.0) |
|  |  |  |
| Decade of Publication |  |  |
| Before 1980 | 1 (2.6) | 121 (0.3) |
| 1980-1989 | 7 (17.9) | 16,074 (39.9) |
| 1990-1999 | 14 (35.9) | 6,483 (16.1) |
| 2000-present | 17 (43.6) | 17,652 (43.8) |

**References**

1. Statistics Canada. (2006) Immigrant Status and Period of Immigration and Place of Birth for the Immigrants and Non-permanent Residents of Canada, Provinces, Territories, Census Metropolitan Areas and Census Agglomerations, 2006 Census. Available: http://www12.statcan.gc.ca/census-recensement/2006/rt-td/index-eng.cfm. Accessed 2011 Aug 01.

2. U.S. Census Bureau. (2009) American Community Survey. Tables S0501 – S0506. Selected Characteristics of the Foreign-Born Population by Region of Birth. Available: http://www.census.gov/acs/www/ Accessed 2011 Aug 01.

3. Statistik Austria. (2010) Bevolkerungsstand 2010 - Population by Citizenship (Table 7). Vienna. Available: http://www.statistik.at/dynamic/wcmsprod/idcplg?IdcService=GET_NATIVE_FILE&dID=85783&dDocName=053629 Accessed 2011 Aug 01.

4. Statistics Belgium. (2011) Population par nationalité, sexe, groupe et classe d'âges au 1er janvier 2010. Available: http://statbel.fgov.be/fr/modules/publications/statistiques/population/population_natio_sexe_groupe_classe_d_ges_au_1er_janvier_2010.jsp Accessed 2011 Aug 20.

5. Czech Statistical Office. (2010) Foreigners in the Czech Republic, 2010. Available: http://www.czso.cz/csu/2010edicniplan.nsf/engp/1414-10 Accessed 2011 Aug 22.

6. Statistics Denmark. (2009) Population by ancestry, region, time, citizenship, age, country of origin and sex. Available: http://www.statbank.dk/statbank5a/default.asp?w=1280 Accessed 2011 Aug 01.

7. Statistics Finland. (2011) Country of birth according to age and gender by region 1990 - 2010. Available: http://pxweb2.stat.fi/database/StatFin/vrm/vaerak/vaerak_en.asp Accessed 2011 Aug 22.

8. Institut national de la statistique et des études économiques. (2006) Recensement de la population - Étrangers selon le sexe, la catégorie de population et la nationalité détaillée. Paris. Available: http://www.insee.fr/fr/bases-de-donnees/default.asp?page=recensements.htm Accessed 2011 Aug 22.

9. Statistisches Bundesamt. (2010) Statistical Yearbook 2010 - Table 2.20. Available: http://www.destatis.de/jetspeed/portal/cms/Sites/destatis/Internet/EN/Navigation/Publications/Crosssection/Yearbook.psml Accessed 2011 Sep 01.

10. Mediterranean Migration Observatory. (2004) Data on immigrants in Greece, from Census 2001, Legalization applications 1998, and valid Residence Permits, 2004 - Table A1. Available: http://www.migrants.gr/?la=5 Accessed 2011 Aug 01.

11. Israel Central Bureau of Statistics. (2008) 2008 Census: Males and Females born abroad by continent of birth, country of birth and age group - Tables 1-10 and 1-11. Available: http://www1.cbs.gov.il/census/census/pnimi_page_e.html?id_topic=11 Accessed 2011 Sep 14.

12. Caritas Italiana. (2010) Dossier Statistico Immigrazione Caritas-Migrantes 2010.

13. Statistics Netherlands. (2011) Population in The Netherlands on 1 January 2011 by sex, age, marital status, origin and generation. The Hague. Available: http://statline.cbs.nl/StatWeb/publication/?VW=T&DM=SLEN&PA=37325eng&LA=EN Accessed 2011 Aug 22.

14. Statistics Norway. (2011) Table 05185 - Foreign born, by sex and country background. Available: http://statbank.ssb.no/statistikkbanken/Default_FR.asp?PXSid=0&nvl=true&PLanguage=1&tilside=selectvarval/define.asp&Tabellid=05185 Accessed 2011 Aug 19.

15. Servico de Estrangeiros e Fronteiras. (2010) Relatorio de Imigracao Fronteiras e Asilo. Available: www.sef.pt Accessed 2011 Aug 19.

16. Central Statistics Office Ireland. (2006) Population usually resident (and present in their usual residence on census night) in each Province and in the Aggregate Town and Aggregate Rural Areas, classified by birthplace.

17. Instituto Nacional de Estadística. (2008) National Immigrant Survey - Revisión del Padrón municipal 2007. Datos a nivel nacional, comunidad autónoma y provincia. Available: http://www.ine.es/en/inebmenu/mnu_migrac_en.htm Accessed 2011 Aug 19.

18. Statistics Sweden. (2011) Foreign-born persons in Sweden by country of birth and sex. Available: http://www.ssd.scb.se/databaser/makro/MainTable.asp?yp=bergman&xu=scb&omradekod=BE&omradetext=Population&lang=2&langdb=2 Accessed 2011 Aug 22.

19. Swiss Federal Institute of Statistics. (2010) Population résidante selon la nationalité par pays, de 1995 à 2009. Available: http://www.bfs.admin.ch/bfs/portal/fr/index/themen/01/07/blank/data/01.html Accessed 2011 Aug 01.

20. BBC News (2008). Born Abroad: An Immigration Map of Britain. Available: http://news.bbc.co.uk/2/shared/spl/hi/uk/05/born_abroad/countries/html/overview.stm Accessed 2011 Aug 19.

21. Australian Bureau of Statistics. (2007) 2006 Census of Population and Housing - Country of Birth of Person (full classification list) by Sex - Catalogue 2068.0. Canberra. Available: http://www.abs.gov.au/websitedbs/d3310114.nsf/home/census+data Accessed 2011 Aug 08.

22. Statistics New Zealand. (2006) 2006 Census of Population and Housing: QuickStats About Culture and Identity, Table 7. Available: http://www.stats.govt.nz/Census/2006CensusHomePage/QuickStats/quickstats-about-a-subject/culture-and-identity.aspx Accessed 2011 Aug 03.

23. Adair R, Nwaneri O (1999) Communicable disease in African immigrants in Minneapolis. Arch Intern Med 159: 83-85.

24. Almog R, Low M, Cohen D, Robin G, Ashkenazi S, et al. (1999) Prevalence of anti-hepatitis A antibodies, hepatitis B viral markers, and anti-hepatitis C antibodies among immigrants from the former USSR who arrived in Israel during 1990-1991. Infection 27: 212-217.

25. Arevalo JA, Arevalo M (1989) Prevalence of hepatitis B in an indigent, multi-ethnic community clinic prenatal population. J Fam Practice 29: 615-619.

26. Aubert JP, Catrice M, Bouee S, Di Pumpo A, Santana P, et al. (2010) [Prevac B: prevention of hepatitis B among migrants from subsaharian Africa and Asia]. [French]. Revue du Prat 60: 13-20.

27. Aweis D, Brabin BJ, Beeching NJ, Bunn JE, Cooper C, et al. (2001) Hepatitis B prevalence and risk factors for HBsAg carriage amongst Somali households in Liverpool. Commun Dis Public Health 4: 247-252.

28. Baldo V, Floreani A, Menegon T, Grella P, Paternoster DM, et al. (2000) Hepatitis C virus, hepatitis B virus and human immunodeficiency virus infection in pregnant women in North-East Italy: a seroepidemiological study. Eur J Epidemiol 16: 87-91.

29. Barry M, Craft J, Coleman D, Coulter HO, Horwitz R (1983) Clinical findings in Southeast Asian refugees. JAMA 249: 3200-3203.

30. Beggio M, Giraldo M, Borella-Venturini M, Mongillo M, Zanetti E, et al. (2007) Prevalence of hepatitis virus A, B, and C markers according to the geographic origin of medical students. [Italian]. Giornale Italiano di Medicina del Lavoro ed Ergonomia 29: 745-747.

31. Ben-Porath E, Hornstein L, Zeldis J, Nahmias J, Gruia M, et al. (1986) Hepatitis B virus infection and liver disease in Ethiopian immigrants to Israel. Hepatology 6: 662-666.

32. Bjerke SE, Vangen S, Holter E, Stray-Pedersen B (2011) Infectious immune status in an obstetric population of Pakistani immigrants in Norway. Scand J Public Health 39: 464-470.

33. Bonura F, Sorgi M, Perna AM, Puccio G, Tramuto F, et al. (2005) Pregnant women as a sentinel population to target and implement hepatitis B virus (HBV) vaccine coverage: a three-year survey in Palermo, Sicily. Vaccine 23: 3243-3246.

34. Bottecchia M, Madejon A, Puente S, Garcia-Samaniego J, Rivas P, et al. (2011) Detection of hepatitis B virus genotype A3 and primary drug resistance mutations in African immigrants with chronic hepatitis B in Spain. J Antimicrob Chemother 66: 641-644.

35. Caruana SR, Kelly HA, De Silva SL, Chea L, Nuon S, et al. (2005) Knowledge about hepatitis and previous exposure to hepatitis viruses in immigrants and refugees from the Mekong Region. Aust N Z J Med 29: 64-68.

36. Catanzaro A, Moser RJ (1982) Health status of refugees from Vietnam, Laos, and Cambodia. JAMA 247: 1303-1308.

37. CDC (2006) Screening for chronic hepatitis B among Asian/Pacific Islander populations--New York City, 2005. MMWR Morb Mortal Wkly Rep 55: 505-509.

38. CDC (1991) Screening for hepatitis B virus infection among refugees arriving in the United States, 1979-1991. MMWR Weekly Rep 40: 784-786.

39. Chadwick RG, Davidson I, Hall AJ (1982) Hepatitis B among Indochinese refugees in Great Britain. Postgrad Med J 58: 676-679.

40. Chaudhary RK, Nicholls ES, Kennedy DA (1981) Prevalence of hepatitis B markers in Indochinese refugees. CMAJ 125: 1243-1246.

41. Chaves NJ, Gibney KB, Leder K, O'Brien DP, Marshall C, et al. (2009) Screening practices for infectious diseases among Burmese refugees in Australia. Emerg Infect Dis 15: 1769-1772.

42. Chemtob D, Fassberg J, Kalka I, Harlap S, Slater PE, et al. (1991) Prevention strategy of hepatitis B virus infection among the Ethiopian Community in Israel. Isr J Med Sci 27: 273-277.

43. Chiaramonte M, Pupo A, Menegon T, Baldo V, Malatesta R, et al. (1998) HBV and HCV infection among non-European Union immigrants in North-East Italy. Epidemiol Infect 121: 179-183.

44. Chironna M, Germinario C, Lopalco PL, Carrozzini F, Barbuti S, et al. (2003) Prevalence rates of viral hepatitis infections in refugee Kurds from Iraq and Turkey. Infection 31: 70-74.

45. Chironna M, Germinario C, Lopalco PL, Quarto M, Barbuti S (2000) HBV, HCV and HDV infections in Albanian refugees in Southern Italy (Apulia region). Epidemiol Infect 125: 163-167.

46. Chironna M, Germinario C, Lupalco PL, Carrozzini F, Quarto M (2001) Prevalence of hepatitis virus infections in Kosovar refugees. Int J Infect Dis 5: 209-213.

47. Christenson B, Bottiger M, Grillner L (1997) The prevalence of hepatitis B in Sweden; a statistical serosurvey of 3381 Swedish inhabitants. Epidemiol Infect 119: 221-225.

48. Dalekos GN, Zervou E, Karabini F, Tsianos EV (1995) Prevalence of viral markers among refugees from southern Albania: increased incidence of infection with hepatitis A, B and D viruses. Eur J Gastroenterol Hepat 7: 553-558.

49. Denburg A, Rashid M, Brophy J, Curtis T, Malloy P, et al. (2007) Initial health screening results for Karen refugees: a retrospective review. Can Commun Dis Rep 33: 16-22.

50. Denis F, Tabaste JL, Ranger-Rogez S (1994) [Prevalence of HBs Ag in about 21,500 pregnant women. Survey at twelve French University Hospitals. The Muticentric Study Group]. [French]. Pathol Biol (Paris) 42: 533-538.

51. Elefsiniotis IS, Glynou I, Brokalaki H, Magaziotou I, Pantazis KD, et al. (2007) Serological and virological profile of chronic HBV infected women at reproductive age in Greece. A two-year single center study. Eur J Obstet Gynec Reprod Biol 132: 200-203.

52. Engebretsen B, Knight A, Shah R (1984) Hepatitis B in Southeast Asian refugees in Iowa. Iowa Med 74: 105-108.

53. Entzel PP, Fleming LE, Trepka MJ, Squicciarini D (2003) The health status of newly arrived refugee children in Miami-Dade County, Florida. Am J Public Health 93: 286-288.

54. Fabris P, Baldo V, Baldovin T, Bellotto E, Rassu M, et al. (2008) Changing epidemiology of HCV and HBV infections in Northern Italy: a survey in the general population. J Clin Gastroenterol 42: 527-532.

55. Faustini A, Franco E, Saitto C, Cauletti M, Zaratti L, et al. (1994) Hepatitis A, B, C and D in a community in Italy of immigrants from NE Africa. J Public Health Med 16: 71-78.

56. Fitzpatrick S, Johnson J, Shragg P, Felice ME (1987) Health care needs of Indochinese refugee teenagers. Pediatrics 79: 118-124.

57. Flatau E, Segol O, Shneour A, Tabenkin H, Raz R (1993) Prevalence of markers of infection with hepatitis B and C viruses in immigrants of operation Solomon, 1991. Isr J Med Sci 29: 387-389.

58. Franco-Paredes C, Dismukes R, Nicolls D, Hidron A, Workowski K, et al. (2007) Persistent and untreated tropical infectious diseases among Sudanese refugees in the United States. Am J Trop Med Hyg 77: 633-635.

59. Friedman SM, DeSilva LP, Fox HE, Bernard G (1988) Hepatitis B screening in a New York City obstetrics service. Am J Public Health 78: 308-310.

60. Garcia-Samaniego J, Soriano V, Enriquez A, Lago M, Martinez ML, et al. (1994) Hepatitis B and C virus infections among African immigrants in Spain. Am J Gastroenterol 89: 1918-1919.

61. Germinario C, Chironna M, Quarto M, Lopalco P, Calvario A, et al. (2000) Immunosurveillance on Kosovar children refugees in Southern Italy. Vaccine 18: 2073-2074.

62. Gish RG, Cooper SL (2011) Hepatitis B in the Greater San Francisco Bay Area: an integrated programme to respond to a diverse local epidemic. J Viral Hepat 18: e40-51.

63. Gjerdingen DK, Lor V (1997) Hepatitis B status of Hmong patients. J Am Board Fam Pract 10: 322-328.

64. Gjorup IE, Skinhoj P, Bottiger B, Plesner AM (2003) Changing epidemiology of HBV infection in Danish children. J Infect 47: 231-235.

65. Glikberg F, Brawer-Ostrovsky J, Ackerman Z (1997) Very high prevalence of hepatitis B and C in Bukharian Jewish immigrants to Israel. J Clin Gastroenterol 24: 30-33.

66. Goldenring JM, Castle GF (1983) Prevalence of disease in Southeast Asian teenagers. Results of screening medical examination at a residential vocational training facility. J Adolesc Health Care 4: 266-269.

67. Goodman RA, Sikes RK (1984) Hepatitis B markers in Southeast Asian refugees. JAMA 251: 27.

68. Hayes EB, Talbot SB, Matheson ES, Pressler HM, Hanna AB, et al. (1998) Health status of pediatric refugees in Portland, ME. Arch Pediatr Adolesc Med 152: 564-568.

69. Hill LL, Hovell M, Benenson AS (1991) Prevention of hepatitis B transmission in Indo-Chinese refugees with active and passive immunization. Am J Prev Med 7: 29-32.

70. Hornstein L, Ben-Porath E, Cuzin A, Baharir Z, Rimon N, et al. (1991) Hepatitis B virus infection in Ethiopian immigrants to Israel. Isr J Med Sci 27: 268-272.

71. Huerga H, Lopez-Velez R (2002) Infectious diseases in sub-Saharan African immigrant children in Madrid, Spain. Pediatric Infect Dis J 21: 830-834.

72. Hurie MB, Gennis MA, Hernandez LV, Dindzans VJ, Davis JP (1995) Prevalence of hepatitis B markers and measles, mumps, and rubella antibodies among Jewish refugees from the former Soviet Union. JAMA 273: 954-956.

73. Hurie MB, Mast EE, Davis JP (1992) Horizontal transmission of hepatitis B virus infection to United States-born children of Hmong refugees. Pediatrics 89: 269-273.

74. Jenista JA, Chapman D (1987) Medical problems of foreign-born adopted children. Am J Dis Child 141: 298-302.

75. Jensen L, Heilmann C, Smith E, Wantzin P, Peitersen B, et al. (2003) Efficacy of selective antenatal screening for hepatitis B among pregnant women in Denmark: Is selective screening still an acceptable strategy in a low-endemicity country? Scand J Infect Dis 35: 378-382.

76. Judson FN, Lince DM, Anders BJ (1984) Health status of Southeast Asian refugees. West J Med 141: 183-188.

77. King K, Vodicka P (2001) Screening for conditions of public health importance in people arriving in Australia by boat without authority. Med J Australia 175: 600-602.

78. Kulstrunk M, Evequoz D, Dubach VC, Banziger W, Lutschg W, et al. (1992) Prevalence of hepatitis B virus in Kurdish refugees. J Hepatol 15: 418-419.

79. Lange WR, Kreider SD, Warnock-Eckhart E (1987) Hepatitis B surveillance in Korean adoptees. Maryland Med J 36: 163-166.

80. Levinne NN, Choong AP (1980) Screening indochinese refugee patients: result of 192 cases. Can Fam Physician 26: 1399-1401.

81. Levy V, Yuan J, Ruiz J, Morrow S, Reardon J, et al. (2010) Hepatitis B sero-prevalence and risk behaviors among immigrant men in a population-based household survey in low-income neighborhoods of northern California. J Immigr Minor Health 12: 828-833.

82. Lifson AR, Thai D, O'Fallon A, Mills WA, Hang K (2002) Prevalence of tuberculosis, hepatitis B virus, and intestinal parasitic infections among refugees to Minnesota. Public Health Rep 117: 69-77.

83. Lin SY, Chang ET, So SK (2007) Why we should routinely screen Asian American adults for hepatitis B: a cross-sectional study of Asians in California. Hepatology 46: 1034-1040.

84. Lopez-Velez R, Huerga H, Turrientes MC (2003) Infectious diseases in immigrants from the perspective of a tropical medicine referral unit. Am J Trop Med Hyg 69: 115-121.

85. Majori S, Baldo V, Tommasi I, Malizia M, Floreani A, et al. (2008) Hepatitis A, B, and C infection in a community of sub-Saharan immigrants living in Verona (Italy). J Travel Med 15: 323-327.

86. Malamitsi-Puchner A, Papacharitonos S, Sotos D, Tzala L, Psichogiou M, et al. (1996) Prevalence study of different hepatitis markers among pregnant Albanian refugees in Greece. Eur J Epidemiol 12: 297-301.

87. Manzardo C, Trevino B, Gomez i Prat J, Cabezos J, Mongui E, et al. (2008) Communicable diseases in the immigrant population attended to in a tropical medicine unit: epidemiological aspects and public health issues. Travel Med Infect Dis 6: 4-11.

88. Martin JA, Mak DB (2006) Changing faces: A review of infectious disease screening of refugees by the Migrant Health Unit, Western Australia in 2003 and 2004. Med J Australia 185: 607-610.

89. Meints L, Chescheir N (2010) Screening for infectious diseases in pregnant, foreign-born women from multiple global areas. J Reprod Med 55: 382-386.

90. Meropol SB (1995) Health status of pediatric refugees in Buffalo, NY. Arch Pediatr Adolesc Med 149: 887-892.

91. Milionis C (2010) Serological markers of Hepatitis B and C among juvenile immigrants from Albania settled in Greece. Eur J General Pract 16: 236-240.

92. Museru OI, Vargas M, Kinyua M, Alexander KT, Franco-Paredes C, et al. (2010) Hepatitis B virus infection among refugees resettled in the U.S.: high prevalence and challenges in access to health care. J Immigr Minor Health 12: 823-827.

93. Museru O, Franco-Paredes C (2009) Epidemiology and clinical outcomes of hepatitis B virus infection among refugees seen at a U.S. travel medicine clinic: 2005-2008. Travel Med Infect Dis 7: 171-174.

94. Nahmias J, Greenberg Z, Berger SA, Hornstein L, Bilgury B, et al. (1993) Health profile of Ethiopian immigrants in Israel: an overview. Isr J Med Sci 29: 338-343.

95. Nelson KR, Bui H, Samet JH (1997) Screening in special populations: a "case study" of recent Vietnamese immigrants. Am J Med 102: 435-440.

96. Ooi WW, Gallagher A, Chen LH (2006) Immunity to hepatitis A and hepatitis B in Indian and Chinese immigrants seen in a travel clinic in Massachusetts, United States. J Travel Med 13: 212-218.

97. Palumbo E, Scotto G, Faleo G, Cibelli DC, Saracino A, et al. (2007) Prevalence of HBV-genotypes in immigrants affected by HBV-related chronic active hepatitis. Arq Gastroenterol 44: 54-57.

98. Palumbo E, Scotto G, Faleo G, Cibelli DC, Angarano G (2007) Prevalence of HBV genotypes in South American immigrants affected by HBV-related chronic active hepatitis. Braz J Infect Dis 11: 311-313.

99. Palumbo E, Scotto E, Cibelli DC, Faleo G, Saracin A, et al. (2008) Immigration and hepatitis B virus: Epidemiological, clinical and therapeutic aspects. Eastern Medit Health J 14: 784-790.

100. Panagopoulos P, Economou A, Kasimi A, Spyropoulou P, Kanellopoulos N, et al. (2004) Prevalence of hepatitis B and C in the maternity department of a Greek district hospital. J Matern Fetal Neonatal Med 16: 106-110.

101. Papaevangelou V, Hadjichristodoulou C, Cassimos D, Theodoridou M (2006) Adherence to the screening program for HBV infection in pregnant women delivering in Greece. BMC Infect Dis 6.

102. Parenti DM, Lucas D, Lee A, Hollenkamp RH (1987) Health status of Ethiopian refugees in the United States. Am J Public Health 77: 1542-1543.

103. Patel PA, Voigt MD (2002) Prevalence and interaction of hepatitis B and latent tuberculosis in Vietnamese immigrants to the United States. Am J Gastroenterol 97: 1198-1203.

104. Perez-Molina JA, Herrero-Martinez JM, Norman F, Perez-Ayala A, Monge-Mahillo B, et al. (2011) Clinical, epidemiological characteristics and indications for liver biopsy and treatment in immigrants with chronic hepatitis B at a referral hospital in Madrid. J Viral Hepat 18: 294-299.

105. Pottie K, Janakiram P, Topp P, McCarthy A (2007) Prevalence of selected preventable and treatable diseases among government-assisted refugees: Implications for primary care providers. Can Fam Physician 53: 1928-1934.

106. Ranger S, Mounier M, Denis F, Alain J, Baudet J, et al. (1990) [Prevalence of hepatitis B (Hbs Ag, Hbe Ag, DNA) and delta virus markers, in about 10,000 pregnant women in Limoges (France)]. [French]. Pathol Biol (Paris) 38: 694-699.

107. Rein DB, Lesesne SB, O'Fallon A, Weinbaum CM (2010) Prevalence of hepatitis B surface antigen among refugees entering the United States between 2006 and 2008. Hepatology 51: 431-434.

108. Roberts NS, Copel JA, Bhutani V, Otis C, Gluckman S (1985) Intestinal parasites and other infections during pregnancy in Southeast Asian refugees. J Reprod Med 30: 720-725.

109. Roudot-Thoraval F, Kouadja F, Wirquin V, Thiers V, Avons P, et al. (1989) [Prevalence of HBs antigen carriers and markers of B virus replication in a population of pregnant women, in France]. [French]. Gastroenterol Clin Biol 13: 353-356.

110. Roussos A, Goritsas C, Pappas T, Spanaki M, Papadaki P, et al. (2003) Prevalence of hepatitis B and C markers among refugees in Athens. World J Gastroenterol 9: 993-995.

111. Saiman L, Aronson J, Zhou J, Gomez-Duarte C, San Gabriel P, et al. (2001) Prevalence of infectious diseases among internationally adopted children. Pediatrics 108: 608-612.

112. Salleras L, Dominguez A, Bruguera M, Plans P, Espunes J, et al. (2009) Seroepidemiology of hepatitis B virus infection in pregnant women in Catalonia (Spain). J Clin Virol 44: 329-332.

113. Sandler SG, Nath N, Biger Y (1977) Seroepidemiology of hepatitis B virus in Israel. Results of a pilot study in Jerusalem. Am J Epidemiol 106: 76-82.

114. Santantonio T, Lo Caputo S, Germinario C, Squarcione S, Greco D, et al. (1993) Prevalence of hepatitis virus infections in Albanian refugees. Eur J Epidemiol 9: 537-540.

115. Sheikh M, Pal A, Wang S, MacIntyre CR, Wood NJ, et al. (2009) The epidemiology of health conditions of newly arrived refugee children: a review of patients attending a specialist health clinic in Sydney. J Paediatrics Child Health 45: 509-513.

116. Skinhoj P, Aldershvile J, Black F, Kjersem H, Kryger P, et al. (1981) Viral hepatitis in southeast Asian refugees. J Med Virol 7: 149-155.

117. Skinhoj P, Aldershvile J, Kjersem M, Black F (1983) Hepatitis B infection in Vietnamese families. J Med Virol 11: 125-129.

118. Skliros E, Lionis C, Foudoulaki L, Sotiropoulos A, Kouroumalis E, et al. (2001) Hepatitis B and C markers in a Kurdish refugee camp in Greece. J Gastroenterol Hepatol 16: 839-840.

119. Skliros EA, Sotiropoulos A, Peppas T, Sofroniadou K, Lionis C (1999) High prevalence of HBV infection markers in refugees from eastern countries. Ital J Gastroenterol Hepat 31: 84-85.

120. Smith A, O'Flanagan D, Igoe D, Cronin J, Forde D, et al. (2000) Outcome of medical screening of Kosovan refugees in Ireland: 1999. Commun Dis Public Health 3: 291-294.

121. Smith MW, Barrett AP, Crewe EB, Griffiths CJ (1984) Serological markers for hepatitis B virus in Indochinese refugees. Aust N Z J Med 14: 171-172.

122. Smith-Garcia T, Brown JS (1989) The health of children adopted from India. J Community Healt 14: 227-241.

123. Stadler LP, Mezoff AG, Staat MA (2008) Hepatitis B virus screening for internationally adopted children. Pediatrics 122: 1223-1228.

124. Stroffolini T, Bianco E, Szklo A, Bernacchia R, Bove C, et al. (2003) Factors affecting the compliance of the antenatal hepatitis B screening programme in Italy. Vaccine 21: 1246-1249.

125. Tafuri S, Prato R, Martinelli D, Melpignano L, De Palma M, et al. (2010) Prevalence of Hepatitis B, C, HIV and syphilis markers among refugees in Bari, Italy. BMC Infect Dis 10.

126. Tiong ACD, Patel MS, Gardiner J, Ryan R, Linton KS, et al. (2006) Health issues in newly arrived Afican refugees attending general practice clinics in Melbourne. Med J Australia 185: 602-606.

127. Tong MJ, Yu M, Co R, Eastin B (1984) Hepatitis B virus markers in the foreign-born Chinese population of Los Angeles, California. J Infect Dis 149.

128. Toro C, Jimenez V, Rodriguez C, Del Romero J, Rodes B, et al. (2006) Molecular and epidemiological characteristics of blood-borne virus infections among recent immigrants in Spain. J Med Virol 78: 1599-1608.

129. Ugwu C, Varkey P, Bagniewski S, Lesnick T (2008) Sero-epidemiology of hepatitis B among new refugees to Minnesota. J Immigr Minor Health 10: 469-474.

130. van Steenbergen JE, Leentvaar-Kuijpers A, Baayen D, Dukers HT, van Doornum GJ, et al. (2001) Evaluation of the hepatitis B antenatal screening and neonatal immunization program in Amsterdam, 1993-1998. Vaccine 20: 7-11.

131. Veldhuijzen IK, van Driel HF, Vos D, de Zwart O, van Doornum GJ, et al. (2009) Viral hepatitis in a multi-ethnic neighborhood in the Netherlands: results of a community-based study in a low prevalence country. Int J Infect Dis 13: e9-e13.

132. Viviano E, Cataldo F, Accomando S, Firenze A, Valenti RM, et al. (2006) Immunization status of internationally adopted children in Italy. Vaccine 24: 4138-4143.
